# H_2_O_2_-producing commensal streptococci disrupt *Streptococcus mutans–Candida albicans* synergism

**DOI:** 10.1128/aem.00333-26

**Published:** 2026-05-20

**Authors:** Hongfei Shao, Hanyeol Yoo, Jian-Na Cai, Hee-Young Jung, Chung-Min Kang, Jae-Gyu Jeon, Dongyeop Kim

**Affiliations:** 1 Department of Preventive Dentistry, School of Dentistry and Institute of Oral Bioscience, Jeonbuk National University 26714, Jeonju, Republic of Korea; 2 Department of Oral Biology, School of Stomatology, Binzhou Medical University 698075, Yantai, People's Republic of China; 3 Department of Pediatric Dentistry, College of Dentistry, Yonsei University 65513, Seoul, Republic of Korea; Centers for Disease Control and Prevention, Atlanta, Georgia, USA

**Keywords:** biofilm, microbial interaction, hydrogen peroxide, synergism, antagonism

## Abstract

Microbial homeostasis is maintained by the antagonistic capacity of commensal bacteria against cariogenic pathogens. In the oral cavity, commensal *Streptococcus*, dominant colonizers of the tooth surface, can produce hydrogen peroxide (H_2_O_2_), modulating virulent cross-kingdom biofilm formation. To investigate their ecological role, clinical isolates from dental plaque were compared with reference strains, including *Streptococcus oralis* ATCC 35037 and *S. oralis* subsp. *tigurinus* J22 to determine their H_2_O_2_-producing capabilities. The antagonistic potential of *S. oralis* against *Streptococcus mutans* and *Candida albicans* was evaluated using microbial and biochemical assessments. In a saliva-coated hydroxyapatite disc model, *S. oralis* strains were co-cultured with *S. mutans* and *C. albicans*. A high H_2_O_2_-producing *S. oralis* J22 inhibited EPS formation in *S. mutans* and yeast-to-hypha transition in *C. albicans*, thereby reducing EPS-mediated bacterial–fungal cell colocalization. Time-lapse confocal imaging revealed that *S. oralis* J22 dominated the biofilm through H_2_O_2_-mediated antagonistic interactions. In contrast, the inhibitory effect of *S. oralis* strains lacking the *spxB* gene on cross-kingdom biofilms was significantly reduced. These data provide ecological insights into how physicochemical properties of early colonizing commensals shape the structure and virulence of cross-kingdom oral biofilm through antimicrobial-mediated antagonistic activity.

**IMPORTANCE:**

The co-existence of *S. mutans* and *C. albicans* accelerates the development of severe early childhood caries, particularly under frequent sucrose exposure. This study demonstrates that early colonizing and antimicrobial-producing oral commensal bacteria can disrupt these pathogenic interactions by modulating their physicochemical associations. These findings highlight the potential of enhancing commensal bacteria as part of novel caries prevention strategies. Further characterization of the functional oral microbiota, especially clinically relevant oral commensals, could advance the development of diagnostic biomarkers and microbiome-targeted therapeutics to prevent painful and costly oral diseases.

## INTRODUCTION

Oral biofilms are complex microbial communities embedded in an extracellular matrix dominated by exopolysaccharides (EPS), which provide structural integrity and a protective barrier. Among environmental factors, dietary sucrose plays a central role in dental caries pathogenesis by fueling EPS synthesis and accelerating acid production. Through constant sugar catabolism, *Streptococcus mutans* rapidly assembles an EPS-rich matrix and acidifies the local microenvironment, promoting enamel demineralization and tooth decay (1–3). Frequent sucrose exposure not only enhances *S. mutans* growth but also disrupts the ecological balance of the oral microbiome, selecting for acidogenic and acid-tolerant species (4, 5).

Microbial interactions within these communities are dynamic and diverse, encompassing antagonistic, cooperative, and synergistic relationships that shape spatial organization and ecological functions (1, 2, 6, 7). Notably, synergistic interactions between *S. mutans* and *Candida albicans* contribute to the development of early childhood caries (ECC), a severe form of tooth decay, which predominantly affects underprivileged preschool children who are highly infected with *S. mutans* and frequently exposed to a sugar-rich diet (8). Although the tooth surface is not considered a natural habitat for *C. albicans*, *S. mutans* facilitates its adherence and colonization through the activity of glucosyltransferase B (GtfB) (9, 10). This enzyme binds to fungal hyphae and synthesizes insoluble glucans in the presence of sucrose, reinforcing bacterial–fungal co-adhesion (11, 12). Such synergistic interactions between *S. mutans* and *C. albicans* enhance metabolic cooperation and virulence, leading to highly cariogenic biofilms characterized by acidogenicity, cross-feeding, and spatially integrated microcolonies (13, 14).

In contrast, antagonistic interactions mediated by commensal bacteria are essential for maintaining microbial homeostasis. Early colonizing oral commensal bacteria, including *S. oralis*, can produce specific antimicrobials, such as hydrogen peroxide (H_2_O_2_) and bacteriocins, which can inhibit pathogen overgrowth and stabilize the oral ecosystem (15–17). H_2_O_2_ production is closely linked to the metabolic activities of the oral commensal bacteria (18). Importantly, previous studies have shown that oral commensal bacteria can inhibit cariogenic pathogens by producing H_2_O_2_ (19), as *S. mutans* is among the H_2_O_2_-susceptible bacteria (20). Although commensal *S. oralis* can modulate the colonization of *S. mutans* (21) and infection of *C. albicans* in the mucosa (22, 23), it remains unclear whether commensal bacteria can alter the infection and biofilm formation of both *S. mutans* and *C. albicans*. Moreover, the specific mechanism of action of how H_2_O_2_-producing *S. oralis* affects the physicochemical associations within *S. mutans–C. albicans* biofilms is largely unknown.

To address these gaps, we employed a cross-kingdom biofilm model that mimics the ecological plaque hypothesis, accounting for the impact of sucrose concentration on microbial interactions. The role of H_2_O_2_-producing oral commensal bacteria in *S. mutans–C. albicans* cross-kingdom biofilm formation and the relevant physicochemical interactions were evaluated by analyzing chemical factors, transcriptomic changes in virulence genes, and biofilm formation, as well as by dissecting the spatial structures of biofilms. This study provides ecological insights into how early colonizing commensals contribute to oral microbial homeostasis by antagonizing synergistic bacterial–fungal interactions.

## RESULTS

### Characteristics of clinical samples

**Fig 1:**
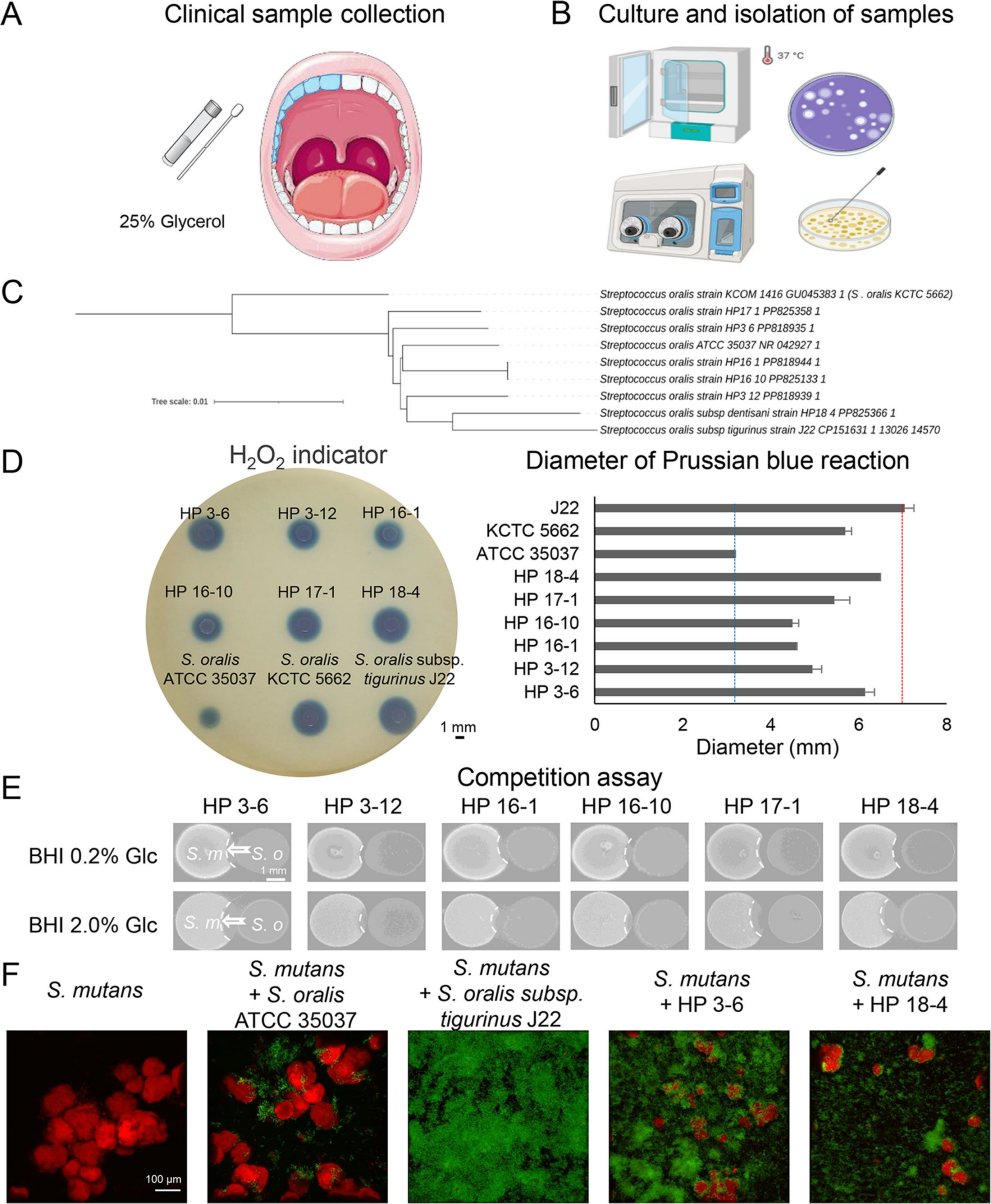
Experimental workflow for isolation, identification, and functional characterization of oral *Streptococcus* isolates. (**A**) Supragingival dental plaque biofilm samples were aseptically collected from the tooth surfaces of participants using sterile curettes or swabs. Dental plaque was preserved in 25% glycerol before processing to maintain microbial viability. (**B**) Samples were incubated on blood agar and selective culture media under various conditions, then streaked onto solid culture media to obtain pure isolates for further analysis. (**C**) Phylogenetic analysis of *S. oralis* isolates. A phylogenetic tree constructed from nucleotide sequences showing the genetic relationships among clinical isolates and reference strains, including *S. oralis* ATCC 35037, *S. oralis* KCTC 5662, and *S. oralis* subsp. *tigurinus* J22. (**D**) Functional characterization of clinical isolates, including their ability to secrete H_2_O_2_, with diameter measurement on H_2_O_2_ indicator agar. (**E**) Competitive assays against *S. mutans* were performed on BHI agar containing different glucose concentrations. Clinical isolates exhibiting high H_2_O_2_ production were selected for further analysis and co-cultured with *S. mutans* and reference strains. (**F**) Biofilm architecture and microbial interactions were subsequently evaluated by confocal microscopy. In the co-culture of *S. mutans* and *C. albicans* with different oral streptococci, *S. mutans*, *S. oralis*, and *C. albicans* are depicted in red, green, and cyan, respectively.

Clinical plaque samples were collected from the enamel surfaces of the maxillary labial and buccal regions (distal to the gingival margin) of patients and preserved in 25% glycerol solution (Fig. 1A). These samples were cultured and isolated using multiple types of culture media—including blood agar, Mitis-Salivarius agar (MSA), and Mitis-Salivarius-Bacitracin agar (MSB)—under different incubation conditions (at 37°C, under 5% CO_2_ or N_2_/CO_2_/H_2_ [85/10/5%]; Fig. 1B).

Phylogenetic analysis based on 16S rRNA gene sequencing revealed that the clinical isolates clustered within the *Streptococcus oralis* group, showing close similarity to reference strains *S. oralis* ATCC 35037 and *S. oralis* subsp. *tigurinus* J22 (Fig. 1C). Despite their high genetic similarity, phenotypic characterization revealed marked variability among isolates in their ability to produce H_2_O_2_ on Prussian blue indicator agar and inhibit *S. mutans* growth on brain heart infusion (BHI) agar plates (Fig. 1D). The confocal imaging of the isolated strain co-cultured with *S. mutans* showed results consistent with the competitive analysis (Fig. 1E and F). Notably, isolates that produce higher levels of H_2_O_2_ exhibited stronger antagonistic activity against *S. mutans*, consistent with the profile observed in the high H_2_O_2_-producing reference strain *S. oralis* J22 (Fig. S1). These findings suggest that even within closely related phylogenetic groups, functional diversity among oral commensal streptococci contributes to differences in ecological competitiveness and biofilm-inhibition potential (24).

### Inhibitory effects of commensal streptococci on *S. mutans* and *C. albicans* in co-culture

**Fig 2:**
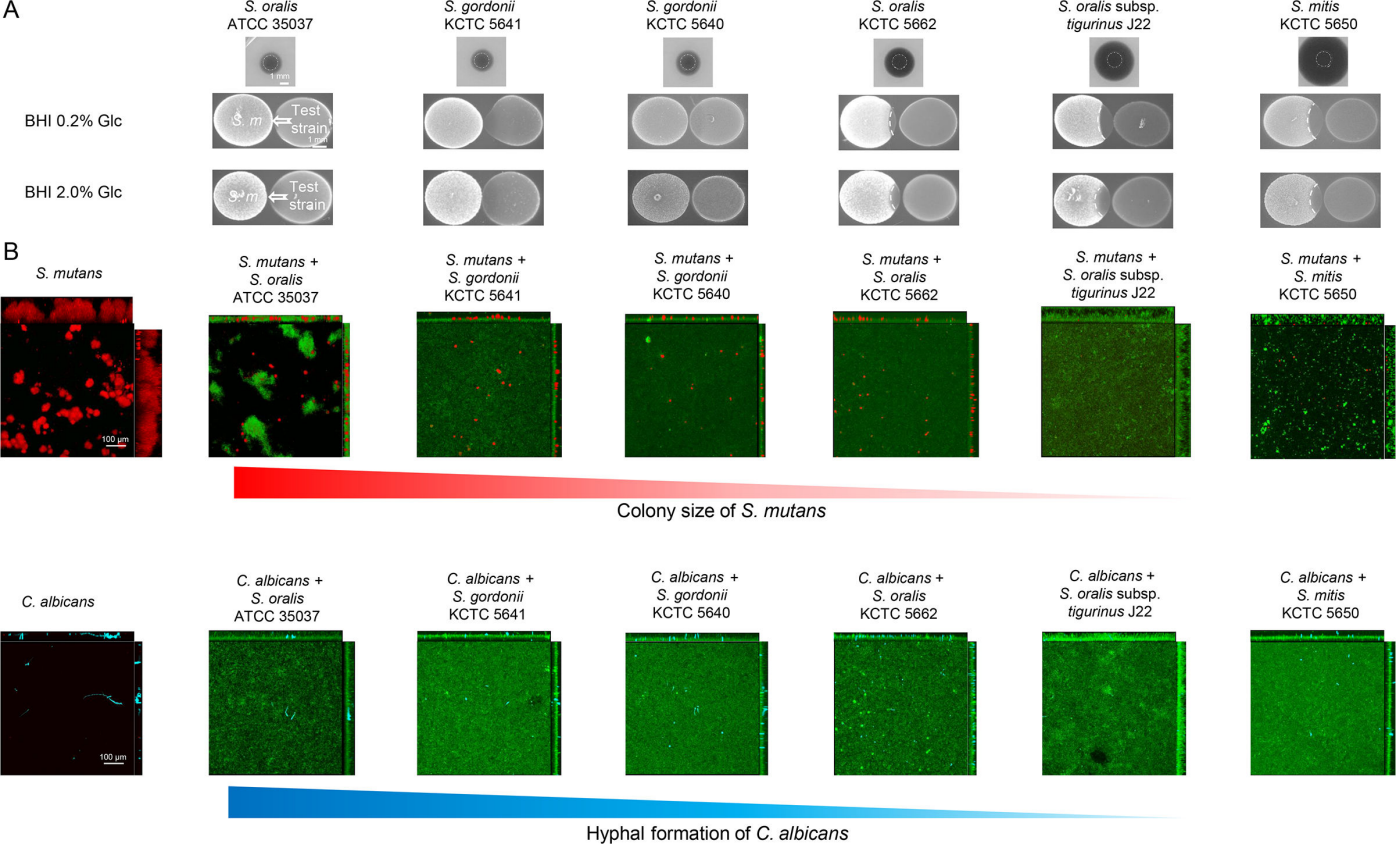
Competitive interactions between oral streptococci and cariogenic pathogens in dual-species biofilms. (**A**) Prussian blue reaction zone indicates relative H_2_O_2_ production (upper panel). Competition assay (lower panel) was conducted between *S. mutans* and oral streptococci under different glucose conditions. (**B**) Confocal imaging of dual-species biofilms. In the co-culture of *S. mutans* and *C. albicans* with different oral streptococci, *S. mutans*, *S. oralis*, and *C. albicans* are depicted in red, green, and cyan, respectively. An increased size of the Prussian blue reaction zone was associated with enhanced inhibition of *S. mutans* colonization and suppression of *C. albicans* hyphal development.

**Fig 3:**
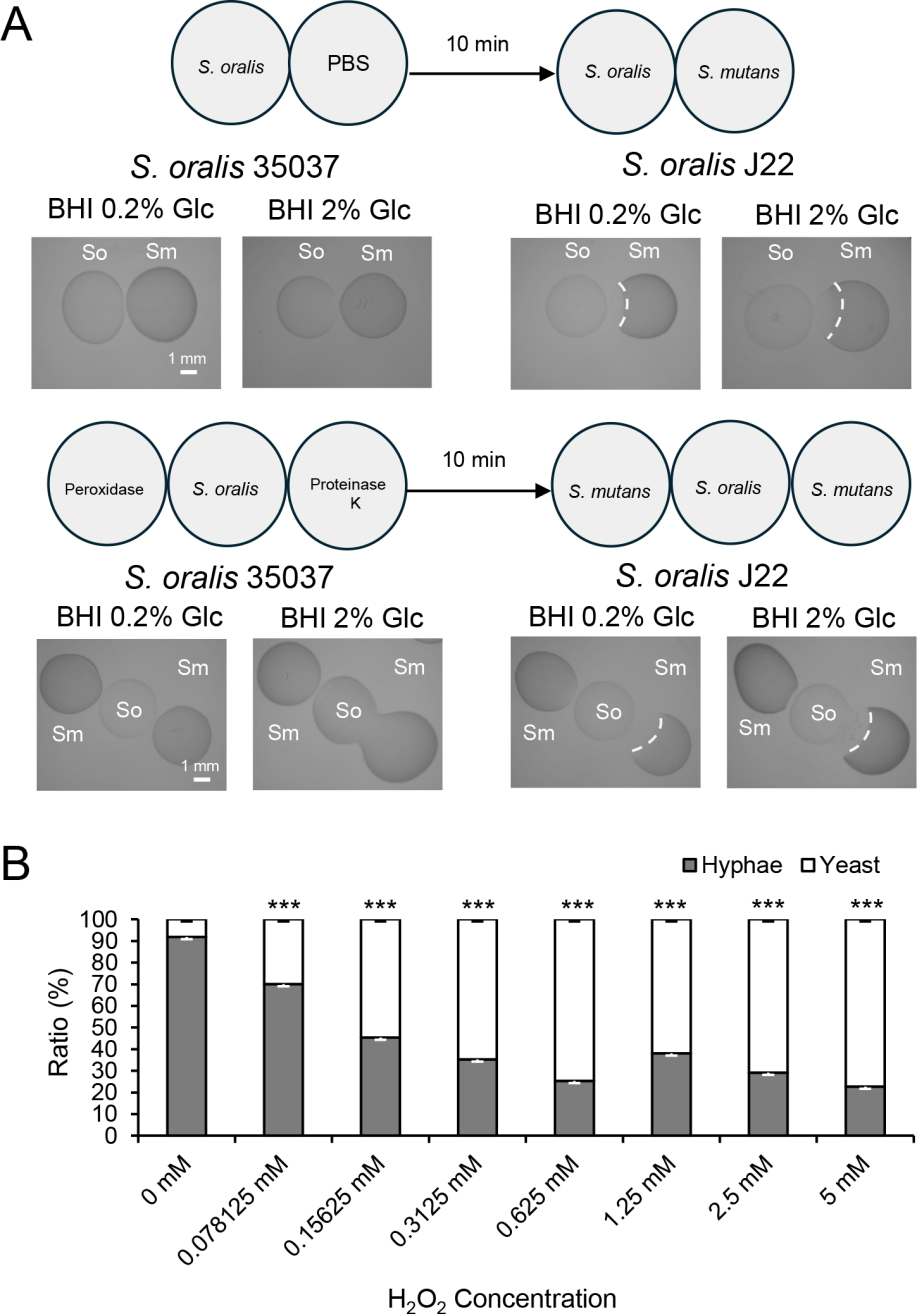
Hydrogen peroxide plays a key role in competing with pathogenic microorganisms. (**A**) A competition assay between *S. oralis* and *S. mutans* on a BHI agar plate. *S. oralis* J22 has a stronger competitive inhibition against *S. mutans*. The effects of peroxidase and proteinase K on the competitive inhibition with *S. mutans* are due to the H_2_O_2_. (**B**) A germ tube assay of *C. albicans. C. albicans* were counted using a hemocytometer under the optical microscope. Based on morphology, the ratio of yeast and hyphae in *C. albicans* was calculated. ***P < 0.001.

In addition to clinical isolates, several reference strains derived from the oral cavity were found to produce H_2_O_2_ at varying levels. Compared with clinical isolates, these reference strains were used for further mechanistic assessment. H_2_O_2_ production by oral commensal streptococci was assessed using Prussian blue indicator agar, in which the diameter of the blue reaction zone serves as a semi-quantitative proxy for relative H_2_O_2_ output. In competitive assays on BHI agar against *S. mutans*, varying levels of H_2_O_2_ production resulted in different levels of inhibitory activity (Fig. 2A). Strains exhibiting larger reaction zones consistently showed stronger inhibitory effects on *S. mutans* colonization and *C. albicans* hyphal formation, indicating a graded association between H_2_O_2_ production and antagonistic activity. Confocal imaging of single-species biofilms revealed that commensal streptococci with higher H_2_O_2_-producing capacity were associated with reduced *S. mutans* microcolony formation and decreased *C. albicans* hyphal development (Fig. 2B). This observation was further supported by quantitative analysis of hyphal formation using a germ tube assay with hemocytometer-based morphological counting (Fig. 3B). Quantitative measurements of the Prussian blue reaction zone diameter on the H_2_O_2_ indicator plate demonstrated that strains producing larger reaction zones exhibited correspondingly larger zones of competitive inhibition against *S. mutans* (Fig. S1). In addition, the inhibitory activity of *S. oralis* J22 against *S. mutans* was maintained under both low- and high-glucose conditions. Notably, although some strains exhibited comparable or higher H_2_O_2_ production, *S. oralis* J22 demonstrated stronger inhibitory activity, likely due to its enhanced surface colonization capacity and ecological competitiveness, suggesting that factors beyond H_2_O_2_ production contribute to antagonistic efficacy.

### High H_2_O_2_-producing *S. oralis* J22 inhibits *S. mutans* growth and *C. albicans* hyphal formation

Using semi-quantitative analysis via *in situ* H_2_O_2_ detection, *S. oralis* J22 was characterized as a high H_2_O_2_-producing bacterium, whereas *S. oralis* ATCC 35037 produced a low level of H_2_O_2_. These variable H_2_O_2_ levels were also found in both the reference strains and clinical isolates from *in vivo* dental plaque samples. In the H_2_O_2_-production assay, *S. oralis* J22 released concentrations exceeding 0.1 mM within 1 h of cultivation and accumulated above 0.2 mM regardless of the sugar concentration during biofilm cultivation (Fig. S2A). High H_2_O_2_ production reflects the metabolic activity of each strain, which is influenced by its cellular redox processes during aerobic metabolism (Fig. S2B). *S. oralis* J22 showed higher metabolic activity at different time points (1, 6, and 18 h) compared to *S. oralis* ATCC 35037 (Fig. S2B).

In the competition assays, *S. oralis* J22 inhibited *S. mutans*, whereas *S. oralis* ATCC 35037 showed no inhibitory effect. In addition, protease and peroxidase treatments confirmed that the inhibitory effect was mediated by H_2_O_2_, but not by protein-based antimicrobial agents such as bacteriocins (19). These findings indicate that *S. oralis* J22 can release significant amounts of H_2_O_2_, maintaining a threshold level to inhibit *S. mutans* under high-sugar conditions (25) (Fig. 3A). The dimorphism of *C. albicans*, particularly the yeast-to-hypha transition, is a unique feature for cross-kingdom biofilm formation, in which hyphae serve as key structural components of EPS-mediated bacterial–fungal association and biofilm development (12). Using biologically relevant concentrations of H_2_O_2_ generated by commensal bacteria, the physiological inhibitory effect of exogenous H_2_O_2_ on the yeast-to-hypha transition of *C. albicans* was determined using a germ tube assay (Fig. 3B). The hyphal proportion decreased in a dose-dependent manner, with a significant reduction observed at 0.3125–0.625 mM H_2_O_2_, which is a biologically relevant concentration in the vicinity of *S. oralis* (Fig. S3).

### Commensal oral streptococci modulate *S. mutans–C. albicans* symbiotic interactions and physical associations

**Fig 4:**
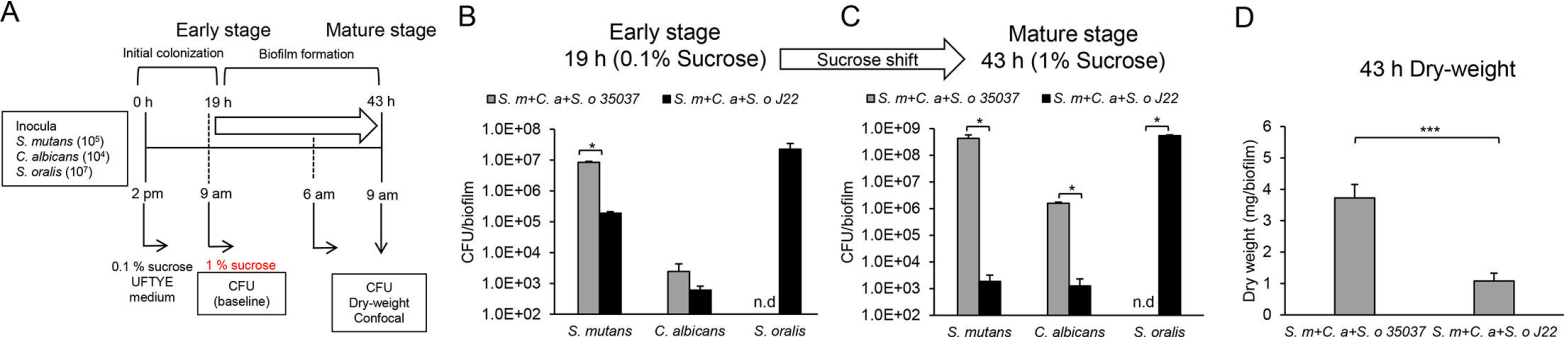
Schematic model for cross-kingdom biofilm formation under ecological sugar shift and the impact of co-existence of high H_2_O_2_-producing *S. oralis* J22. (**A**) Microorganisms are cultured in UFTYE medium supplemented with sucrose at low (0.1% wt/vol; equivalent to ~3 mM) or high (1% wt/vol; ~29 mM) concentrations to simulate dietary sugar–driven ecological shifts in the oral environment, and biofilms are formed on hydroxyapatite discs. (**B**) After 19 h, the biofilms on the hydroxyapatite discs are collected, separated by ultrasound, and smeared on blood culture medium for viable cell counting. (**C**) After 43 h, the biofilms on the hydroxyapatite discs are collected, and viable cell counts and 3D architecture of the biofilm are assessed. Panels B and C represent early-stage (19 h, 0.1% sucrose) and mature-stage (43 h, 1% sucrose) biofilms, modeling a sucrose-driven ecological shift. Comparisons across panels highlight the effect of increasing sucrose on microbial interactions, while differences within each panel reflect strain-dependent effects. (**D**) The biofilms are collected, centrifuged, and freeze-dried, and the dry weight is measured. The data were subjected to a *t*-test for pairwise comparison. NS, not significant. *P < 0.05; **P < 0.01; ***P < 0.001.

To evaluate whether H_2_O_2_-producing *S. oralis* can inhibit the physicochemical associations between *S. mutans* and *C. albicans*, *S. oralis* was co-cultured in a cross-kingdom biofilm model. Biofilm samples were collected at the early (19 h) and mature (43 h) stages of culture to assess the regulatory effect of *S. oralis* on the formation of *S. mutans* microcolonies and the EPS matrix, as well as the colony growth of *C. albicans* (Fig. 4A). Despite differences in multispecies biofilm accumulation, *S. oralis* ATCC 35037 and *S. oralis* J22 showed comparable early-stage single-species attachment and initial colonization under identical inoculum conditions (Fig. S4). Increasing sucrose concentration promoted the synergistic interaction between *S. mutans* and *C. albicans*, as evidenced by enhanced microbial growth and biofilm development in the presence of *S. oralis* ATCC 35037 (Fig. 4B). In contrast, *S. oralis* J22 effectively disrupted this sucrose-driven shift, significantly reducing *S. mutans* viability at both 19 and 43 h and limiting the expansion of the cross-kingdom biofilm (Fig. 4C). Although *C. albicans* cell counts showed modest variation with increasing sucrose, the overall expansion of the cross-kingdom biofilm remained limited in the presence of *S. oralis* J22 (Fig. 4B and C). Consistent with these findings, total biofilm biomass was reduced in the presence of *S. oralis* J22 compared to those with *S. oralis* ATCC 35037 (Fig. 4D).

Notably, while *S. oralis* ATCC 35037 failed to maintain viability under these conditions and became undetectable at the mature biofilm stage, *S. oralis* J22 persisted and maintained its antagonistic activity. These results indicate that although sucrose enhances pathogenic synergism, high H_2_O_2_-producing commensals such as *S. oralis* J22 can counteract this effect and preserve ecological balance under cariogenic conditions.

To validate the direct contribution of H_2_O_2_, exogenous H_2_O_2_ was added to the cross-kingdom biofilm model at defined concentrations. Increasing H_2_O_2_ levels resulted in dose-dependent reductions in viable cell counts and total biofilm biomass, without major shifts in medium pH at biologically relevant concentrations (Fig. S5). These findings support a threshold-dependent inhibitory effect of H_2_O_2_ on biofilm development.

**Fig 5:**
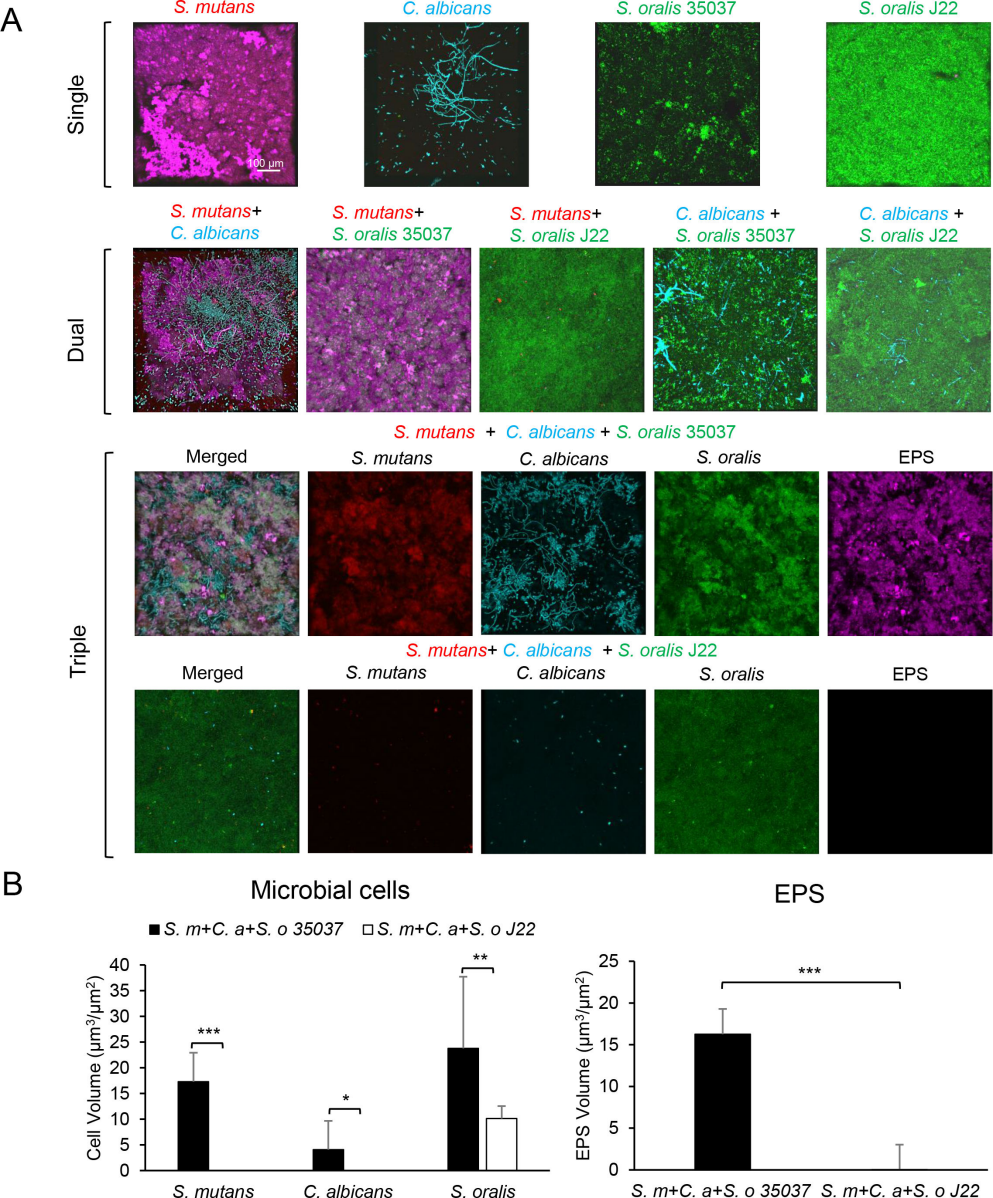
*S. oralis* J22 can inhibit physical associations between *S. mutans–C. albicans* interactions. (**A**) Confocal imaging of single-, dual-, and triple-species biofilms was performed on oral cross-kingdom biofilms cultured on saliva-coated hydroxyapatite under ecological conditions for 43 h. *S. mutans* is shown in red, *C. albicans* in cyan, *S. oralis* in green, and EPS in magenta. Confocal images show the 3D structure of the triple-species biofilm and are split into separate channels to display the marker signals of the different strains. (**B**) Computational analysis quantifies the total biovolume and EPS biovolume of the 43-hour triple-species biofilm images. The data were subjected to a *t*-test for pairwise comparison. *P < 0.05; **P < 0.01; ***P < 0.001.

Physical associations between microbial cells and the EPS matrix in cross-kingdom biofilms were analyzed using confocal imaging and quantitative image analysis. 3D-rendered confocal images revealed morphological changes in *C. albicans* hyphae, *S. mutans* microcolonies, and EPS formation (Fig. 5A). *S. mutans* and *C. albicans* form a robust biofilm with an EPS-rich matrix encapsulating both the cells within the microcolonies, where *C. albicans* exists as yeast or filamentous hyphae. In contrast, when co-cultured with *S. oralis* J22, significant reductions in *S. mutans* colonization, EPS production, and *C. albicans* hyphal formation were observed. *S. oralis* J22 maintained dominance by producing high levels of H_2_O_2_, exhibiting a strong antagonistic effect at 43 h. Quantitative imaging analysis showed that the total biomass and EPS production in the three-species biofilm were significantly reduced when co-cultured with *S. oralis* J22 compared to *S. oralis* ATCC 35037 (Fig. 5B).

### H_2_O_2_ is a key factor in modulating cross-kingdom biofilm formation

**Fig 6:**
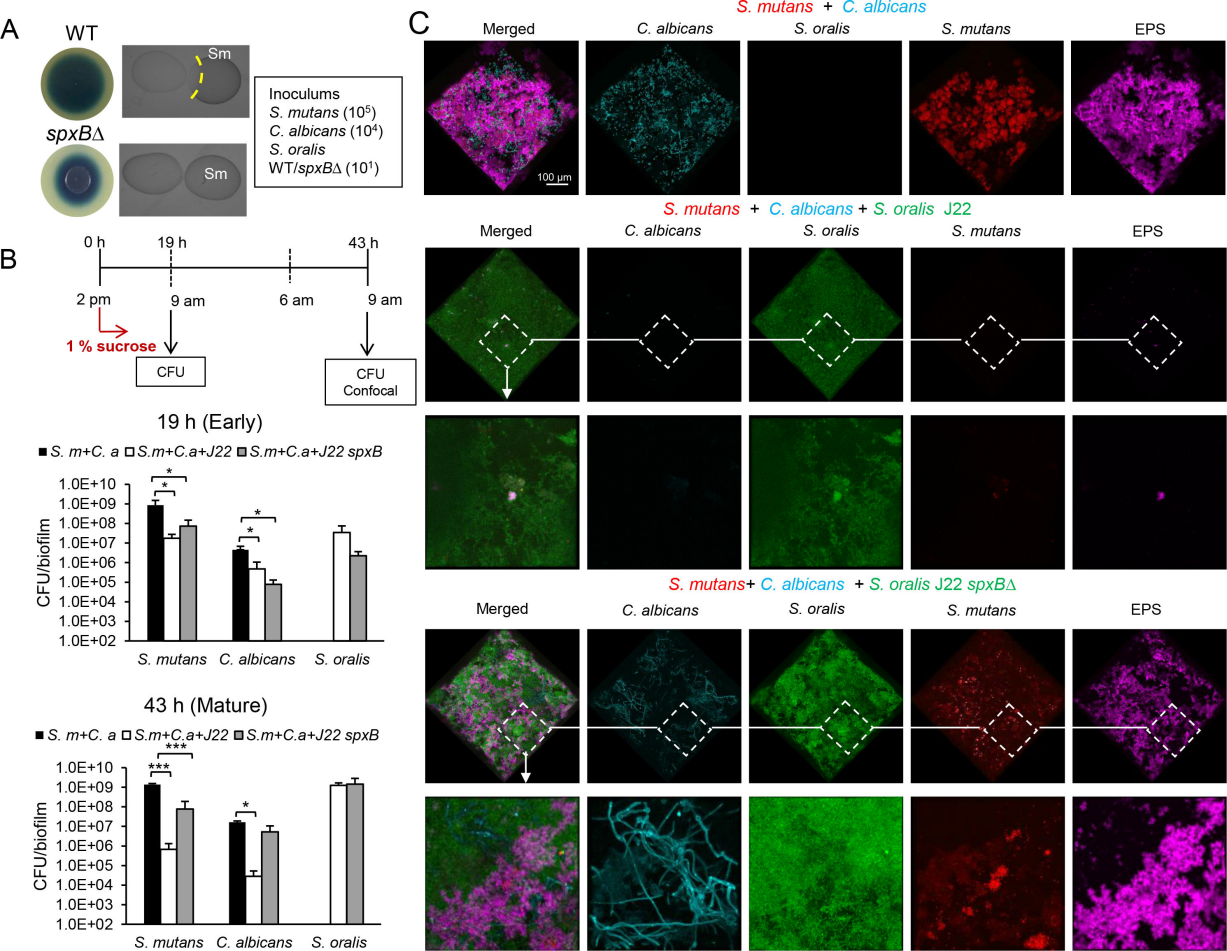
Comparison between *S. oralis* wild-type and *spxB* mutant strains. (**A**) Semi-quantification of H_2_O_2_ production from *S. oralis* J22 WT and *S. oralis* J22 *spxB* strains on H_2_O_2_ indicator agar and competition assay between *S. oralis* J22 WT and *S. oralis* J22 *spxB* against *S. mutans*. (**B**) Experimental design of cross-kingdom biofilm with/without *S. oralis* J22 in a saliva-coated hydroxyapatite model. Viable cell counts of cross-kingdom biofilm with *S. oralis* on blood agar at 43 h were measured. The data were subjected to a *t*-test pairwise comparison. *P < 0.05; ***P < 0.001. (**C**) Confocal images of *S. mutans–C. albicans–S. oralis* dual- and triple-species biofilm are acquired at 43 h. Detailed imaging, including zoomed-in views, clearly depicts the altered physical associations between bacteria and fungi.

To assess whether H_2_O_2_ is the primary factor influencing cross-kingdom biofilm formation, the *spxB* mutant strain of *S. oralis* J22 was used. The *spxB* mutant produced significantly less H_2_O_2_ than the wild-type strain (Fig. 6A), resulting in a reduced ability to inhibit the *S. mutans* growth. Quantitative analysis further revealed that both metabolic activity and H_2_O_2_ production were significantly reduced in the *spxB* mutant (Fig. S2), correlating with its reduced competitive fitness in cross-kingdom biofilms (Fig. 6A).

In the saliva-coated hydroxyapatite (sHA) disc model, *S. oralis* J22 exhibits strong competitive activity. Preliminary experiments indicated that high inoculum levels of this strain resulted in near-complete suppression of cross-kingdom biofilm development at early time points, precluding meaningful comparison of antagonistic mechanisms. Therefore, the initial inoculum of *S. oralis* was deliberately reduced to allow assessment of graded inhibitory effects. Although the *spxB* mutant still produced measurable levels of H_2_O_2_ (approximately 0.2 mM at 6 h), exceeding those of *S. oralis* ATCC 35037, it failed to exert a significant inhibitory effect on cross-kingdom biofilm formation, as assessed by viable cell counts and total biomass. Initial inoculum sizes were verified by CFU enumeration at 0 h to ensure experimental consistency. These results indicate that reduced inoculum conditions enabled discrimination between partial H_2_O_2_ production and functionally effective antagonism (Fig. 6B). Confocal imaging confirmed that the *spxB* mutant exhibited only limited inhibitory effects on *S. mutans* microcolony formation and failed to suppress *C. albicans* hyphal development compared with the wild-type strain. Although the *spxB* mutant partially reduced *S. mutans* microcolonies relative to the coculture lacking *S. oralis*, it did not inhibit hyphal formation and, in some cases, was associated with increased hyphal abundance. Despite exhibiting higher viable cell counts in 43-hour-old biofilms, the *spxB* mutant lacked the robust antagonistic activity observed in the wild-type strain. Together, these findings indicate that reduced H_2_O_2_ production in the *spxB* mutant limits its ability to disrupt cross-kingdom biofilm architecture, supporting a key role for *spxB* in *S. oralis* J22 and highlighting H_2_O_2_ as a key contributor within a multifactorial regulatory framework governing bacterial–fungal interactions within cross-kingdom biofilms (Fig. 6C).

### H_2_O_2_ plays a crucial role in regulating EPS formation during cross-kingdom biofilm formation

**Fig 7:**
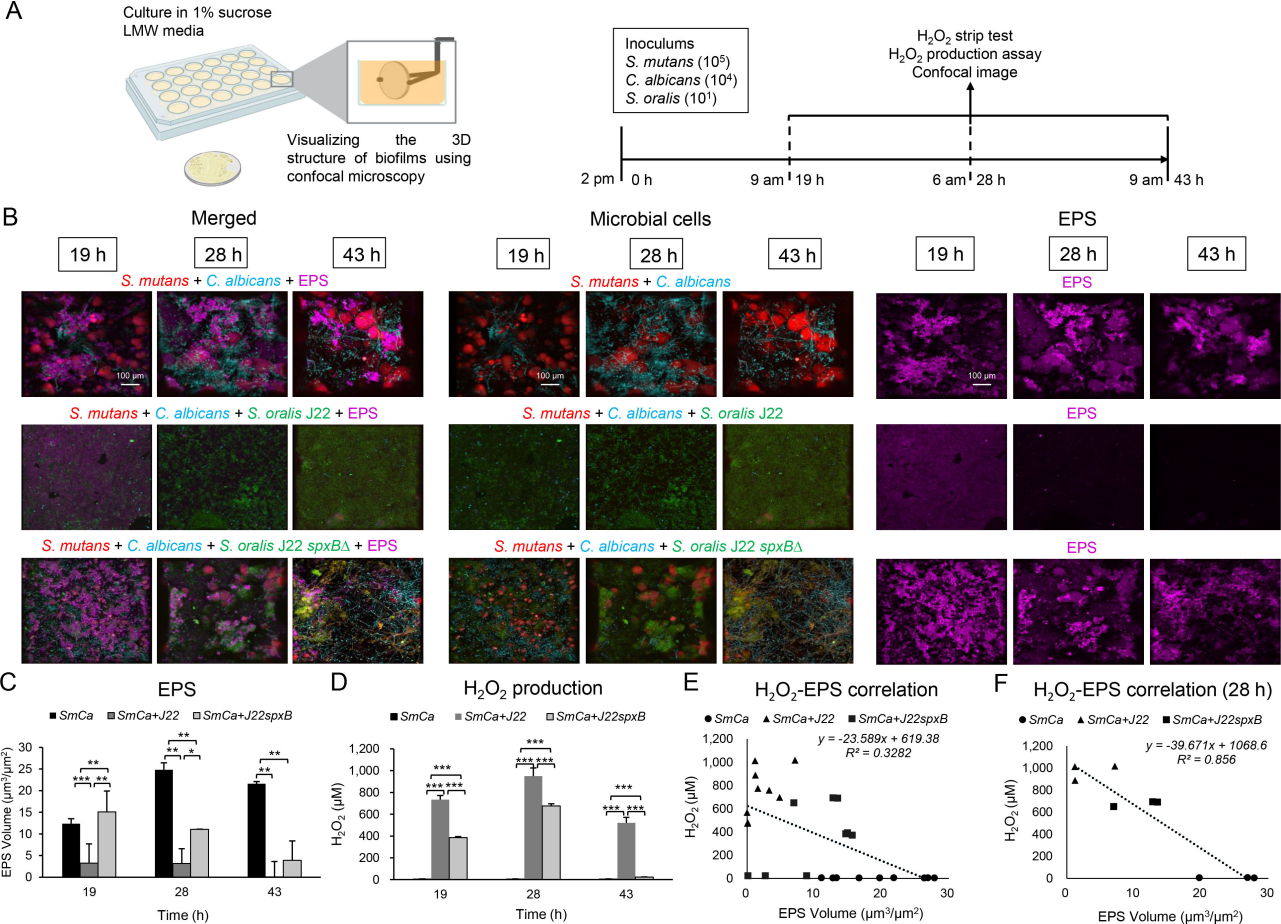
Time-dependent assessment of *S. oralis* J22 on changes in cross-kingdom biofilm structure and its H_2_O_2_ production. (**A**) Biofilms were cultured in UFTYE medium supplemented with 1% sucrose. Biofilm architecture was visualized using confocal laser scanning microscopy at three developmental stages (19 h, 28 h, 43 h). H_2_O_2_ production was quantified using an enzymatic assay. (**B**) Representative three-dimensional reconstructions reveal temporal differences in biofilm composition. (**C**) Quantitative image analysis of EPS biovolume. (**D**) Quantitative analysis of H_2_O_2_ production. (**E**) H_2_O_2_-EPS correlation. EPS biovolume was significantly reduced in biofilms containing wild-type *S. oralis* J22 compared to other groups. (**F**) H_2_O_2_-EPS correlation at 28 h. H_2_O_2_ production was highest in wild-type *S. oralis* J22 co-cultures and showed a strong negative correlation with EPS accumulation at 28 h (*R*² = 0.856). The data were subjected to a *t*-test for pairwise comparison. *P < 0.05; **P < 0.01; ***P < 0.001.

Quantitative relationships between accumulated H_2_O_2_ levels and EPS inhibition were further examined using enzymatic assays and correlation analyses. To investigate the role of H_2_O_2_ produced by *S. oralis* in modulating EPS production within *S. mutans–C. albicans* cross-kingdom biofilms, *S. oralis* was introduced into a multispecies biofilm model. Biofilms and culture media were sampled at early (19 h), intermediate (28 h), and mature (43 h) stages (Fig. 7A). Confocal imaging revealed the 3D architecture of the biofilms and enabled quantification of EPS production (Fig. 7B). Quantitative image analysis showed that the wild-type J22 strain, which secretes high levels of H_2_O_2_, markedly suppressed EPS production in the cross-kingdom biofilm (Fig. 7C). In contrast, the *spxB* mutant strain exhibited significantly lower H_2_O_2_ accumulation in the culture medium and diminished its inhibitory activity against EPS formation (Fig. 7D). Correlation analysis across the three time points revealed a negative correlation between H_2_O_2_ levels produced by the commensal bacterium and EPS biovolume in the cross-kingdom biofilm. Specifically, decreased H_2_O_2_ concentration correlated with reduced suppression of EPS synthesis (Fig. 7E). Notably, at 28 h, a pronounced negative correlation was observed (*R*² = 0.856) (Fig. 7F). H_2_O_2_ strip assays further validated these findings by confirming lower H_2_O_2_ concentrations in corresponding culture conditions (Fig. S6). These results underscore the critical function of the *spxB* gene in modulating H_2_O_2_ production and affirm the substantial impact of H_2_O_2_ on EPS regulation in cross-kingdom biofilms.

### The presence of high H_2_O_2_-producing *S. oralis* suppressed the expression of EPS production- and microbial fitness-related genes in *S. mutans*

To elucidate the role of high H_2_O_2_-producing bacteria in the virulence of cross-kingdom biofilms, quantitative gene expression analysis targeting virulence factors was conducted. Gene expression analysis revealed that H_2_O_2_ produced by *S. oralis* J22 downregulated EPS formation and microbial fitness-related genes in *S. mutans*. Specifically, *gtfB* and *gtfC* genes encoding glucosyltransferase B and C (GtfB and GtfC), which are crucial for the synthesis of insoluble glucans (1, 3), were significantly downregulated (Fig. S7). Since glucans facilitate biofilm formation and bacterial adhesion, repressed gene expression can be associated with reduced biofilm formation and cell-EPS cohesion. Furthermore, *atpD* encoding the β-subunit of ATP synthase (26) and *ldh* encoding lactate dehydrogenase (LDH), which are involved in sugar metabolism and acid production, were notably repressed (Fig. S7), indicating an impact on energy metabolism (26, 27). Competence-associated genes, *comA* related to competence-stimulating peptide (CSP) precursor synthesis, and *comC* encoding CSP, which regulates quorum sensing, genetic transformation, biofilm formation, and bacteriocin production (28), were also downregulated. Additionally, *nlmA* and *nlmB* encode the subunits of bacteriocin and are also downregulated in co-culture with *S. oralis* J22. Notably, when co-cultured with the *spxB* mutant strain, the expression levels of EPS production- and microbial fitness-related genes were restored to the levels observed in dual *S. mutans–C. albicans* biofilms. In *C. albicans*, the expression of genes related to hyphal formation (*ALS3* and *HYR1*) and the metabolic mechanisms (*SOD4* and *STP2*) was evaluated. The results showed no significant changes in *SOD4* and *STP2* regardless of the presence or absence of *spxB* in *S. oralis* J22. However, the expression of hypha-related genes increased in co-culture with *S. oralis* J22, suggesting that the complex regulation of fungal morphogenesis in *C. albicans* may lead to compensatory overexpression of *ALS3* and *HYR1*, even in the absence of changes in transcription factors or the oxidative stress response pathway. The Cek1 MAPK pathway, an important signal transduction pathway in *C. albicans*, primarily responds to cell wall stress, osmotic stress, mating pheromones, and certain surface signals that promote hyphal growth. Due to the presence of strains with high H_2_O_2_ production, the expression of these related genes was suppressed (Fig. S7). This finding suggests that elevated local oxidative stress may interfere with upstream signaling required for stable hyphal commitment, thereby uncoupling hypha-associated gene expression from morphological outcomes (29). Together, these data indicate that H_2_O_2_-mediated antagonism does not simply repress fungal gene expression globally, but instead perturbs regulatory signaling networks governing morphogenesis, leading to transcriptional compensation without effective hyphal development. Further studies assessing pathway activation, post-transcriptional regulation, and fungal oxidative stress tolerance will be required to fully resolve these mechanisms.

## DISCUSSION

A highly diverse microbial community plays a critical role in maintaining oral and systemic health. For example, a well-balanced microbiome supports essential functions, such as nutrient metabolism, immune modulation, and colonization resistance against pathogens. However, microbial dysbiosis is often associated with oral diseases, including dental caries, when the microbial diversity is disrupted by unbalanced dietary habits that act as environmental stressors (1).

Microbial interactions within the oral cavity shape the composition and function of the microbiome (1, 30). Synergistic microbial interactions, such as the association between *S. mutans* and *C. albicans*, enhance oral biofilm virulence and contribute to disease progression, including ECC (1, 12, 14). In contrast, antagonistic interactions are crucial in modulating microbial diversity and maintaining ecological balance (1, 19, 30). In the present study, *S. oralis* exhibited potent antagonistic activity against *S. mutans* and *C. albicans* through H_2_O_2_ production, disrupting EPS-mediated cross-kingdom biofilm formation by inhibiting *S. mutans* EPS formation and *C. albicans* hyphal formation. These findings highlight how microbial competition contributes to the regulation of biofilm structure and virulence, reinforcing the importance of antagonistic commensal-pathogen interactions in oral ecosystems (31). Importantly, the antagonistic effects described here reflect local, strain-specific interactions observed within a defined *in vitro* cross-kingdom biofilm model.

Rather than functioning as conventional probiotics intended for exogenous administration, H_2_O_2_-producing streptococci are better viewed as endogenous ecological stabilizers. Their antagonistic activity appears to act locally, constraining the expansion of pathobionts and reducing virulent cross-kingdom interactions within specific micro-niches (30). As such, their contribution to eubiosis relies on preserving or supporting existing commensal communities rather than direct supplementation.

Given the polymicrobial niches of the oral cavity, the key question is how commensal bacteria support and maintain a healthy microbial community through their intrinsic antagonistic properties. Environmental factors, including pH, carbohydrate availability, and oxygen gradients, are expected to strongly influence SpxB-dependent H_2_O_2_ production and, consequently, the magnitude of antagonistic effects (17). Consistent with prior work, antagonism mediated by H_2_O_2_ is strongly dose-dependent and influenced by community context. Previous studies have shown that *S. mutans* exhibits a dose-dependent response to H_2_O_2_, with biofilm-associated cells requiring higher levels of oxidative stress to impair growth or fitness than planktonic cells (32). Similarly, H_2_O_2_ can promote hyphal formation in *C. albicans* at low or transient concentrations; however, this effect is highly context-dependent (33). In our system, sustained exposure to biologically relevant H_2_O_2_ levels (~0.3–0.6 mM), comparable to those produced by *S. oralis*, was associated with suppression of hyphal development, indicating a shift from morphogenetic stimulation to oxidative inhibition. In cross-kingdom biofilms, this tolerance is further enhanced by catalase activity produced by *C. albicans*, which can protect *S. mutans* from oxidative stress (34). Consistent with these observations, although the *spxB* mutant of *S. oralis* J22 produced higher levels of H_2_O_2_ than *S. oralis* ATCC 35037, its H_2_O_2_ output remained insufficient to reach the inhibitory threshold required to disrupt *S. mutans-C. albicans* biofilms. In contrast, wild-type J22 produced H_2_O_2_ levels exceeding this threshold, resulting in sustained inhibition of cross-kingdom biofilm development. Notably, the *spxB* mutant did not exhibit a complete loss of viability at later time points, despite reduced H_2_O_2_ production. This contrasts with the behavior of low H_2_O_2_-producing strains such as *S. oralis* ATCC 35037, suggesting that factors beyond H_2_O_2_ contribute to ecological fitness. Furthermore, increased expression of biofilm-related genes observed in the *spxB* mutant may reflect compensatory regulatory responses under reduced oxidative stress. Together, these findings indicate that H_2_O_2_ is a key, but not exclusive, determinant of antagonistic interactions, operating within a broader network of metabolic and regulatory mechanisms.

H_2_O_2_-mediated inhibition in oral biofilms is likely to be highly localized rather than uniformly distributed. Mitis group streptococci preferentially occupy oxygenated niches near the biofilm surface, where SpxB-dependent H_2_O_2_ production is maximized (35). As a result, oxidative antagonism may primarily affect neighboring cells within diffusion-limited microenvironments rather than the entire community. Furthermore, the introduction of additional taxa into complex biofilms can buffer or spatially redistribute antagonistic interactions, allowing mutual persistence of otherwise competing species. For example, *S. sanguinis* and *S. mutans* can coexist in polymicrobial communities despite H_2_O_2_-mediated antagonism (36), highlighting the importance of spatial structure and community composition in shaping ecological outcomes (2, 30, 35).

Additionally, nutrient availability, especially that of fermentable carbohydrates, modulates microbial community functions. In *Streptococcus sanguinis* and *Streptococcus gordonii*, both *spxB* expression and H_2_O_2_ production were suppressed by carbon catabolite repression via catabolite control protein A (CcpA) (18). Among various sugars, sucrose acts as a key modulator of H_2_O_2_ production and microbial competition. High-sucrose concentrations diminished H_2_O_2_ production in *S. gordonii* (19), thereby attenuating its antagonistic potential. In contrast, *S. oralis* J22 maintained its antagonistic activity against *S. mutans* even under high-sucrose conditions. These findings suggest that bacterial communities capable of sustaining H_2_O_2_ production without strong sugar-mediated repression may more effectively inhibit pathogenic bacterial–fungal associations, even under cariogenic conditions, such as high and frequent sucrose exposures. Emerging evidence also suggests that *Streptococcus mitis* exhibits a similar phenotype, characterized by potent H_2_O_2_ production and strong inhibitory effects on cariogenic pathogens (37). However, while H_2_O_2_-producing commensals can play a protective role in maintaining microbial balance, it is important to recognize that exceeding certain thresholds of H_2_O_2_ concentration may negatively affect the surrounding normal flora and disrupt ecological stability (38).

Based on these observations, we propose a conceptual model that highlights antagonistic commensal bacterial interactions to reduce the virulence of cross-kingdom biofilm. This concept may be applied to the development of novel strategies to prevent and manage biofilm-associated diseases. Specifically, accelerating the growth of antimicrobial agent-producing bacteria through dietary modulation could be a potential candidate for oral health-promoting strategies. For instance, dietary magnesium can affect *spxB* transcription and abundance in both *S. sanguinis* and *S. gordonii*, thereby promoting the production of H_2_O_2_ by oral commensal bacteria and inhibiting biofilm formation (39).

Despite these insights, this study has limitations, as the evidence is derived from *in vitro* models. Although prior work has demonstrated concordance between *in vitro* antagonistic interactions and *in vivo* caries outcomes (19), further validation using *in situ*, *ex vivo*, or animal models will be required to determine how community composition, oxygen tension, and dietary fluctuations shape redox-mediated antagonism under physiologically relevant conditions. In particular, future studies incorporating direct measurements of cellular redox state, metabolic flux, and organic acid profiles will be necessary to resolve the relative contributions of SpxB-dependent pathways beyond H_2_O_2_ production. Integrative multi-omics approaches may further elucidate the regulatory networks underlying commensal antagonism and microbial resilience (40). Moreover, a functional understanding of commensal community dynamics, particularly their capacity for antagonism, may serve as a key diagnostic indicator of oral health status (41). To this end, strain-specific effects contributing to variations in the antagonistic activity of commensal bacteria can be elucidated through comparative whole-genome analysis. This approach enables the identification of genetic determinants associated with functional traits of commensal bacteria, including their capacity to inhibit pathobionts. Recognizing and characterizing these microbial interactions is critical for developing microbiome-targeted therapies to maintain oral homeostasis and prevent disease progression.

Collectively, this research illustrates the contrasting ecological and functional states of the oral microbiome, distinguishing a cariogenic microenvironment associated with dysbiosis from a healthy microenvironment characterized by eubiosis. In the cariogenic state, *S. mutans* and the hyphal form of *C. albicans* interact synergistically within an acidic, EPS-rich environment, promoting microbial co-adhesion, enhanced fitness, biofilm virulence, and enamel demineralization. In contrast, the healthy state is shaped by antagonistic interactions mediated by H_2_O_2_-producing commensals. These bacteria suppress cariogenic pathogens by limiting EPS synthesis and inhibiting the yeast-to-hypha transition in *C. albicans*, thereby favoring less virulent biofilm architectures. Together, these findings identify H_2_O_2_ as a key metabolic factor influencing cross-kingdom biofilm organization and microbial balance, and suggest its potential utility as a functional indicator of cariostatic conditions and oral health status.

## MATERIALS AND METHODS

### Bacterial strains and culture conditions

*S. mutans* UA159 is a cariogenic microorganism. For inoculum preparation, bacterial cells were grown to an optical density at 600 nm (OD_600_) of 1.0 in ultrafiltered (10 kDa molecular-mass cutoff membrane; Millipore, MA) tryptone-yeast extract broth (UFTYE; 2.5% tryptone and 1.5% yeast extract) with 1% (wt/vol) glucose at 37°C and 5% CO_2_, as described previously (13). *S. oralis* ATCC 35037 and *S. oralis* subsp. *tigurinus* strain J22 (*S. oralis* J22) were used for the competition and biofilm formation assays. H_2_O_2_ production by *S. oralis* ATCC 35037 and *S. oralis* J22 wild-type (WT)/*spxB*-defective mutant (*spxB*:ermAM) strains (gift from Jens Kreth, Oregon Health & Science University) was assessed using brain heart infusion (BHI)/Prussian blue indicator agar plates, as previously described (17, 42). *C. albicans* SC5314 was cultured in UFTYE with 1% (wt/vol) glucose at pH 5.5, where the morphology of *C. albicans* was dominated by its yeast form. Clinical *S. oralis* strains were isolated from plaque samples obtained from the Yonsei University Oral Biobank. A total of 28 isolates were collected from supragingival plaques and cultured in both aerobic and anaerobic environments with 5% CO_2_ and anaerobic environments. Then, the clinical strains were isolated and screened on BHI agar supplemented with 5% (vol/vol) defibrinated sheep blood agar, MSA, and MSB media, respectively. They were then stored in a mixture of 25% glycerol and 1× TSB at −80°C for further experiments. Primers 27F and 1492R are widely used universal primers for amplifying the full-length bacterial 16S rRNA gene for taxonomic identification and diversity studies of clinical isolates. The combination of hexacyanoferrate and ions in aqueous solution yields a blue precipitate of Prussian blue in the presence of H_2_O_2_ via the following sequential reactions: equal densities and volumes of *S. oral* strains were dropped onto H_2_O_2_ indicator agar plates (containing 0.2% glucose) to determine the H_2_O_2_ production capacity by measuring the halo zone diameter. To test the effect of sugar concentration on the inhibitory effect of *S. oralis* against *S. mutans*, *S. oralis* strains isolated from clinical plaque samples were selected and used. The glucose concentration in BHI was also quantified (0.2%), and it is differentiated from the 2% glucose-added group (BHI + 1.8% glucose). In addition, to evaluate the biological characteristics of oral symbiotic bacteria, reference strains representing typical oral commensal bacteria were incorporated into the competitive assay against *S. mutans*. These strains were selected for their prevalence and clinical relevance, providing a more representative model of the native oral microbiota. Each bacterial species was co-cultured with *S. mutans* and *C. albicans* for 24 h under standardized conditions. For biofilm visualization, *S. mutans* UA159 LDH-GFP, a green fluorescent protein (GFP)-tagged strain, was employed in both mono- and dual-species biofilm experiments. Bacterial cells were universally labeled using the EUB338 probe (5′-GCTGCCTCCCGTAGGAGT-3′), while *C. albicans* was specifically stained with Calcofluor White for subsequent confocal laser scanning microscopic imaging. This study involves isolation, identification, and comparison to model strains (potent, high H_2_O_2_ producer) in phylogenetically similar *S. oralis* strains. Based on screening results for oral commensal bacteria isolated from clinical samples and reference strains, H_2_O_2_ plays an important role in interactions among oral microorganisms. *S. oralis* ATCC 35037 and *S. oralis* J22, which have different H_2_O_2_ production capabilities, were selected as typical representatives for subsequent experimental analysis.

### Bioactivity analysis and quantitative H_2_O_2_ production of oral commensal bacteria

Following a comprehensive screening of clinical isolates and reference strains, two highly representative *S. oralis* strains—*S*. *oralis* ATCC 35037 and *S. oralis* J22—were selected for subsequent mechanistic investigations and bioactivity assessments. Colorimetric assays of XTT (2,3-bis-(2-methoxy-4-nitro-5-sulfophenyl)-2H-tetrazolium-5-carboxanilide, Sigma, St. Louis, MO, USA) and H_2_O_2_ were conducted in a 96-well plate to study the biological activity of *S. oralis* and the production of H_2_O_2_ over time under different sugar concentrations. The cell proliferation assay using XTT is a colorimetric assay for the nonradioactive quantification of cellular proliferation, viability, and cytotoxicity (43), based on the reduction of a yellow tetrazolium salt to an orange formazan dye by metabolically active cells. The formazan dye formed was directly quantified using a microplate reader. An H_2_O_2_ quantitative assay kit (ab102500, BioVisiom, Milpitas, CA) was used to measure H_2_O_2_ in biological samples via colorimetric and fluorometric assays. In this assay, horseradish peroxidase (HRP) reacted with a probe and H_2_O_2_ to produce a colored product (λmax = 570 nm) with red fluorescence (Ex/Em = 535/587 nm). The detection limit for H_2_O_2_ in the fluorometric assay was 2 pmol per assay (or 40 nM) (44).

### Competitive spotting and germ tube assays

To assess the contribution of hydrogen peroxide and proteinaceous antimicrobials to *S. oralis*–mediated antagonism, a competitive spotting assay was performed with or without enzymatic treatments, as previously described with minor modifications (19). Briefly, overnight cultures of *S. oralis* strains and *S. mutans* were grown in BHI medium, adjusted to an optical density at 600 nm (OD_600_) of 0.5, and washed once with phosphate-buffered saline (PBS). Five microliters of *S. oralis* culture suspension was spotted onto BHI agar plates supplemented with the indicated carbohydrate concentration. Competing *S. mutans* cultures (5 µL, OD_600_ = 0.5) were spotted adjacent to the *S. oralis* inoculum. To evaluate the contribution of H_2_O_2_, peroxidase (Sigma-Aldrich; 100 U/mL final concentration) was spotted on the agar medium prior to *S. mutans* spotting. To assess the involvement of proteinaceous antimicrobials, proteinase K (Sigma-Aldrich; 100 µg/mL final concentration) was spotted on the agar medium prior to *S. mutans* spotting. Control (PBS) without enzymatic supplementation was included in parallel. After spotting, plates were air-dried for 10 min under aseptic conditions and incubated at 37°C and 5% CO_2_ for 24 h. Antagonistic activity was assessed by measuring the inhibition zone or distortion of *S. mutans* colony morphology adjacent to the *S. oralis* spot. To verify the effects of H_2_O_2_ on the yeast-to-hypha transition in *C. albicans*, various concentrations of H_2_O_2_ were exposed to *C. albicans* during germ tube formation. *C. albicans* (10^5^ cells/mL) was inoculated in sugar-free fetal bovine serum (FBS) culture medium with different concentrations of H_2_O_2_ and cultured at 37°C with 5% CO_2_ for 3 h, then centrifuged at 5,000 × *g* for 5 min (45). The pellet was resuspended in 100 μL FBS solution, and the morphological characteristics at different H_2_O_2_ concentrations were observed under an optical microscope (Zeiss, Oberkochen, Germany) or a confocal microscope (Nikon, Tokyo, Japan). A hemocytometer was used to determine the hyphal formation ratio to the yeast form of *C. albicans*. Hyphal and yeast forms were distinguished morphologically, and at least 200 cells per sample were counted to determine the hyphal proportion.

### Biofilm formation on saliva-coated hydroxyapatite disc model

Biofilms were formed on hydroxyapatite (HA) discs (surface area, 2.7 ± 0.2 cm^2^) vertically suspended in 24-well plates using a custom-made wire specimen holder. Each bacterial suspension was mixed to obtain an inoculum of *S. mutans* (10^5^ CFU/mL), *S. oralis* (10^7^ CFU/mL), and *C. albicans* (10^4^ CFU/mL). Whole saliva was collected to prepare pellicle-coated hydroxyapatite (sHA), mimicking the smooth surfaces of a pellicle-coated tooth. To minimize donor-specific variability and ensure reproducibility, saliva was pooled from multiple healthy donors prior to use.

Pooled whole saliva from multiple donors was used to form a pellicle to ensure experimental consistency and reproducibility. Consistent with the ecological plaque hypothesis, the mixed bacterial population was inoculated into UFTYE containing 0.1% (wt/vol) sucrose (0.00292 mol/L) and then incubated for 19 h to form the initially colonized community on the surface. The initial biofilm was then transferred to UFTYE containing 1% sucrose (0.0292 mol/L) to stimulate a cariogenic challenge at 19 h. The culture medium was changed after 28 h, and the biofilm (43 h) was subjected to viable cell counting, dry weight measurement, and confocal imaging with species-specific labeling (2, 46).

### Confocal microscopy analysis of spatial structure in mixed-species biofilms

Intact and undisturbed biofilms formed on the sHA were analyzed for their three-dimensional (3D) architecture by fluorescence *in situ* hybridization (FISH), as described previously. Bacterial cells were labeled by using species-specific FISH probes: MUT590, 5′-ACTCCAGACTTTCCTGAC-3′ with Cy5 for *S. mutans*; MIT447, 5′-CACCCGTTCTTCTCTTACA-3′ with FAM for *S. oralis*; and *C. albicans* and exopolysaccharides were stained with Calcofluor and labeled by Alexa Fluor 647-dextran conjugates (Invitrogen, Thermo Fisher Scientific, Waltham, MA), respectively (13). The 3D biofilm architecture was acquired using a Nikon C2 plus with a 20× (1.0 numerical aperture). The biofilms were sequentially scanned using diode lasers (405, 488, 561, and 640 nm), and the emitted fluorescence was collected with a multialkali PMT detector (490–550 nm for Alexa Fluor 488, 565–620 nm for Cy3, and 645–700 nm for Cy5 or Alexa Fluor 647). ImageJ and Nikon software were used to create a 3D rendering to visualize the architecture of the biofilms. Computational quantitative analysis of the bacterial cell/EPS biomass was performed using COMSTAT, written as a script for MATLAB version R2023b software (MathWorks, USA) (46).

### Application of *S. oralis* J22 *spxB*-defective strain in cross-kingdom biofilm models

SpxB, which encodes pyruvate oxidase, is responsible for the production of H_2_O_2_ by the metabolic activities of mitis group streptococci (47). The *S. oralis* J22 *spxB* mutant was used to test whether the inhibitory effect of oral commensal bacteria on cross-kingdom biofilms was altered when genes associated with H_2_O_2_ production were deleted. During biofilm formation, *S. oralis* J22 wild-type or *spxB* mutant strains were co-inoculated with 10^5^ *S. mutans* and 10^4^ *C. albicans* in the presence of 1% sucrose. The initial inoculum of *S. oralis* was adjusted (10^1^ *S. oralis* instead of 10^7^ *S. oralis* in the ecological model) to prevent immediate dominance by this highly competitive strain and to enable assessment of threshold-dependent H_2_O_2_-mediated antagonism during early cross-kingdom biofilm development.

### Effects of oral commensal bacterium-derived H_2_O_2_ on EPS formation

sHA discs were cultured in UFTYE supplemented with 1% sucrose. *S. oralis* J22 wild-type and its isogenic *spxB* mutant were co-inoculated with *S. mutans* LDH-GFP (10⁵ CFU) and *C. albicans* (10⁴ CFU) under sucrose-supplemented conditions. To simulate a cariogenic microenvironment, the initial inoculum of *S. oralis* was deliberately reduced to 10¹ CFU, in contrast to the higher colonization levels used in ecological modeling. To examine the correlation between H₂O₂ accumulation and the structural development of biofilms over time, culture medium samples were collected at early (19 h), middle (28 h), and mature (43 h) stages of biofilm formation. For microscopic analysis, *C. albicans* was stained with Calcofluor White (Sigma), while exopolysaccharides (EPS) were labeled using Alexa Fluor 647-conjugated dextran. Universal bacterial detection was achieved with the Cy3-labeled EUB338 probe (5′-GCTGCCTCCCGTAGGAGT-3′). Following image acquisition, the *S. mutans* LDH-GFP signal was computationally subtracted to isolate the Cy3-derived signal corresponding to *S. oralis*. EPS channels were extracted and quantitatively analyzed using MATLAB to assess EPS production within cross-kingdom biofilms at each time point. H_2_O_2_ levels in the culture medium were initially assessed using colorimetric test strips (Sigma-Aldrich, St. Louis, MO), with the *S. mutans–C. albicans* co-culture serving as the negative control lacking endogenous H_2_O_2_ production. This preliminary assessment was followed by precise quantification using an enzymatic H_2_O_2_ assay kit. The two-step approach allowed rapid, point-of-care-like screening of H_2_O_2_ accumulation, followed by accurate measurement for correlation analyses. Together, these assays enabled robust association of H_2_O_2_ levels with the structural and compositional properties of cross-kingdom biofilms, including EPS production.

### Assessment of gene expression via quantitative real polymerase chain reaction (qRT-PCR) analysis

RNA was extracted using protocols optimized for *in vitro* (27) and purified using a RNeasy MinElute Cleanup Kit (Qiagen, Valencia, CA). qRT-PCR was performed to measure gene expression in *S. mutan*s and *C. albicans*. Briefly, cDNAs were synthesized using 0.5 µg of purified RNA and the Bio-Rad iScript cDNA synthesis kit (Bio-Rad Laboratories, Inc., Hercules, CA). The resulting cDNAs were amplified using an Applied Biosystems StepOne system with specific primers for the targeted genes (*gtfB, gtfC, gtfD*, *atpD*, *ldh*, *comA*, *comB*, *nlmA*, and *nlmB* in *S. mutans; ALS3*, *CAP1*, *CEK1*, *EFG1*, *HYR1*, *RAS1*, *SOD4*, and *STP2* in *C. albicans*). Relative gene expression was calculated by normalizing each gene of interest to 16S rRNA, and *DAD1* was used as an internal control for *S. mutans* and *C. albicans*, respectively. The primer sequences used in this study are shown in the supplemental material.

### Statistical analysis

Data are presented as mean ± standard deviation (SD) and were obtained from at least three independent experiments. Data were analyzed using analysis of variance (ANOVA) with *post hoc* Tukey’s test for multiple comparisons, and a pairwise comparison was conducted using Student’s *t*-test. Statistical analyses were performed using SPSS version 26.0 software (IBM, Armonk, NY).

## Data Availability

The 16S rRNA sequences have been deposited in the NCBI GenBank database under the following accession numbers: PP818935.1, *S. oralis* strain HP3-6; PP818939.1, *S. oralis* strain HP3-12; PP818944.1, *S. oralis* strain HP16-1; PP825133.1, *S. oralis* strain HP16-10; PP825358.1, *S. oralis* strain HP17-1; and PP825366.1, *S. oralis* subsp. *dentisani* strain HP18-4.

## SUPPLEMENTAL MATERIAL

Supplemental figures and table aem.00333-26-s0001.docx Fig. S1 to S7 and Table S1.

ASM does not own the copyrights to Supplemental Material that may be linked to, or accessed through, an article. The authors have granted ASM a non-exclusive, world-wide license to publish the Supplemental Material files. Please contact the corresponding author directly for reuse.
