# Supplementary material for: H2O2-producing commensal streptococci disrupt Streptococcus mutans–Candida albicans synergism
Source: Appl Environ Microbiol. 2026 May 20;92(6):e00333-26. doi: 10.1128/aem.00333-26 (PMC13274379; doi:10.1128/aem.00333-26)
Supplement: Supplemental figures and table — Fig. S1 to S7 and Table S1. [file aem.00333-26-s0001.docx]

**Supplementary Information**

**
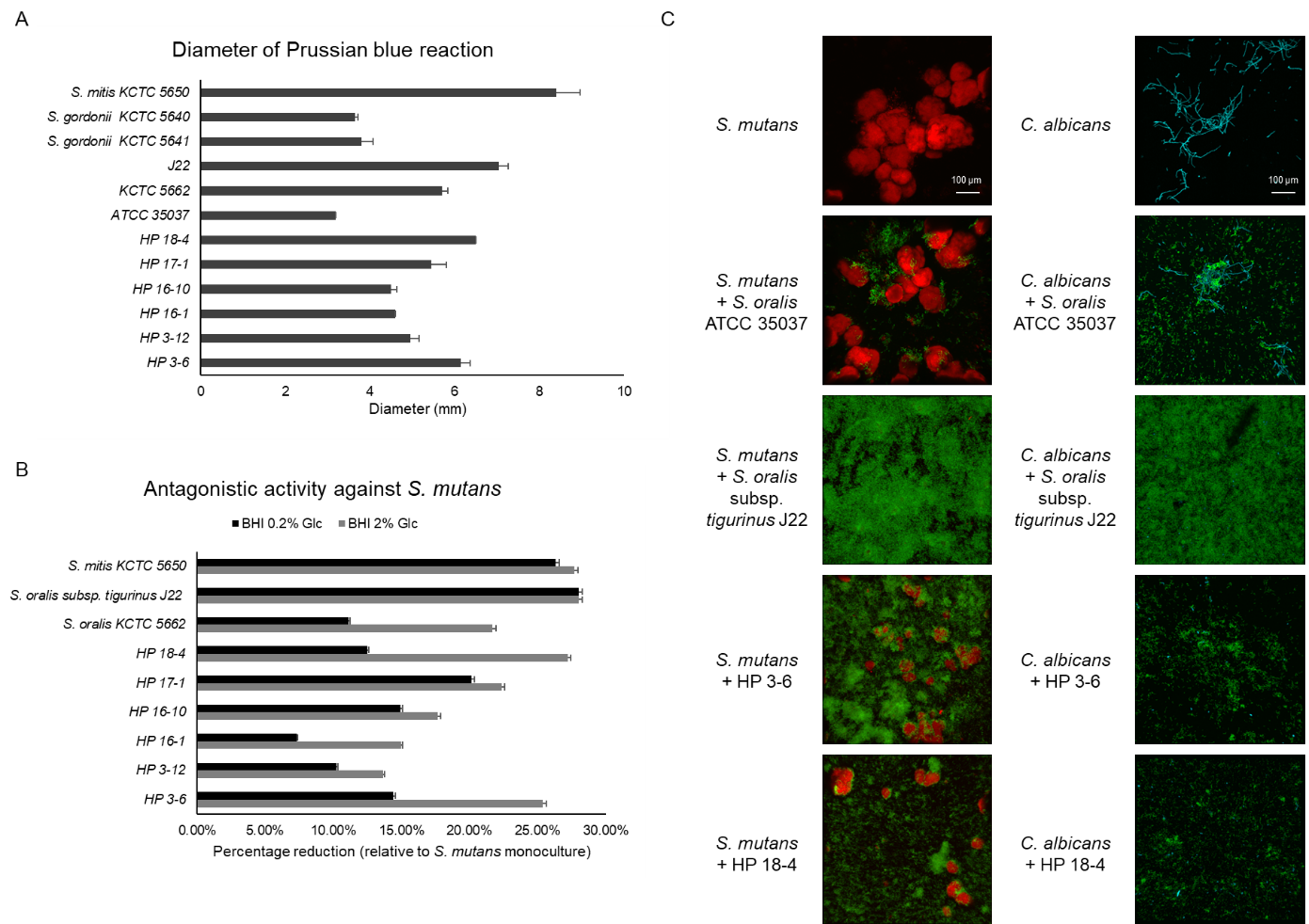
**

**Figure S1. Preliminary H_2_O_2_ screening and competition test of clinical isolates and reference strains*.*** (A) The diameter of the H_2_O_2_ colorimetric reaction produced by the strain cultured in Prussian blue petri dishes was measured. (B) By measuring the diameter, the area of competitive inhibition between the test strain and *S. mutans* was semi-quantitatively calculated. (C) High-yielding H_2_O_2_ clinical isolates and standard strains were selected, and confocal images were obtained from their co-culture with *S. mutans* or *C. albicans*. *S. mutans*, *S. oralis*, and *C. albicans* are depicted in red, green, and cyan, respectively.


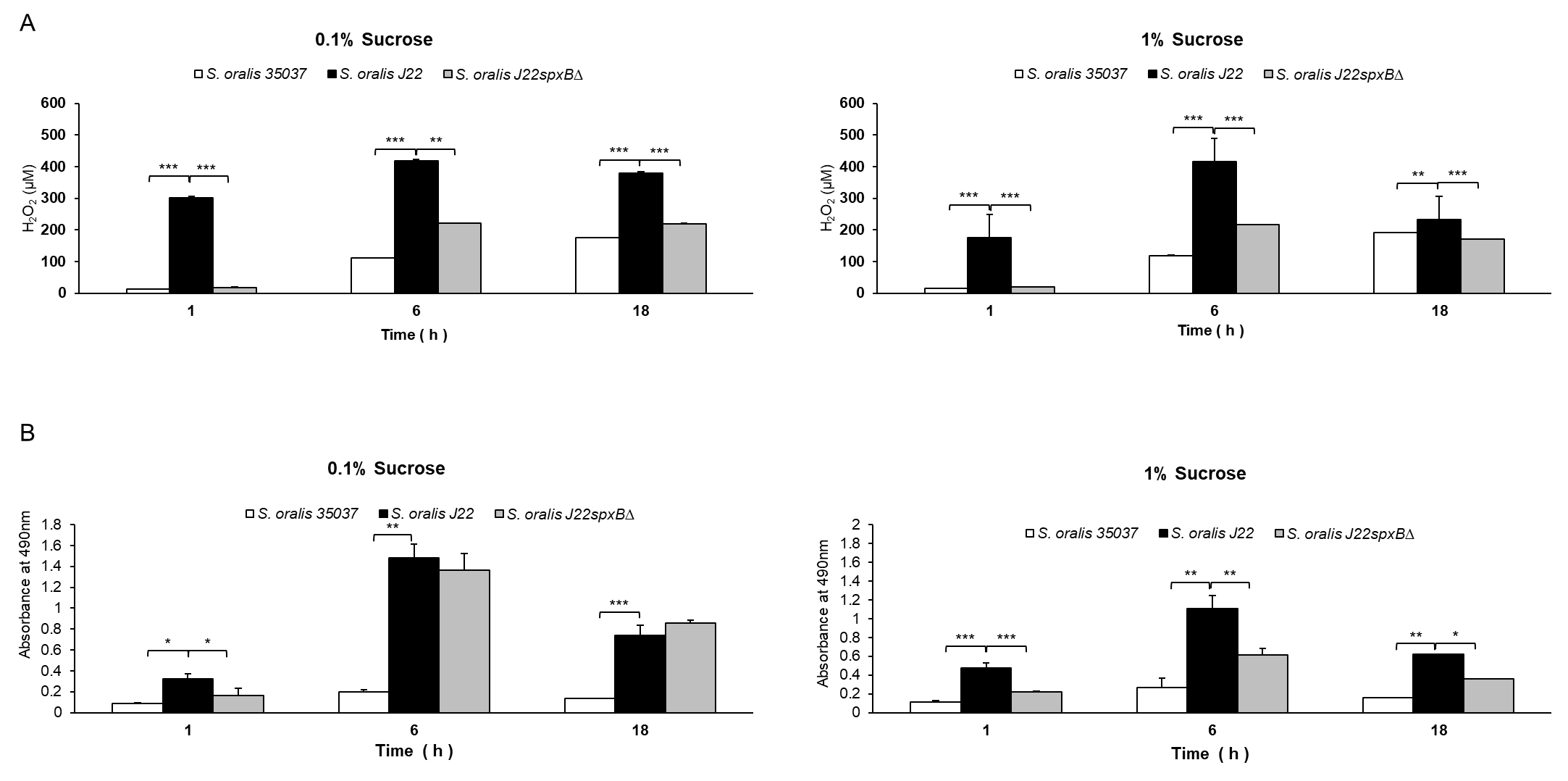


**Figure S2.** **SpxB plays an important role in the metabolism of *S. oralis.*** (A) In the H_2_O_2_ assay, horseradish peroxidase (HRP) reacts with a probe and H_2_O_2_ to produce a product with color (λmax = 570 nm) and red fluorescence (Ex/Em=535/587 nm). The standard curve fits a linear regression (y = 456489x + 5934, *R^2^* = 0.996). Accumulated H_2_O_2_ production of *S. oralis* was monitored for 18 h in the presence of either 0.1% or 1% sucrose. (B) XTT is extracellularly reduced to an orange formazan product in the presence of metabolically active cells. Consequently, the metabolic activity of a microbial culture can be quantified using a visible spectrophotometer in the 430-490 nm range. The metabolic activity was traced for 18 h in the presence of either 0.1% or 1% sucrose. The data were subjected to a *t*-test for pairwise comparison. *p < 0.05; **p < 0.01, ***p < 0.001.

**
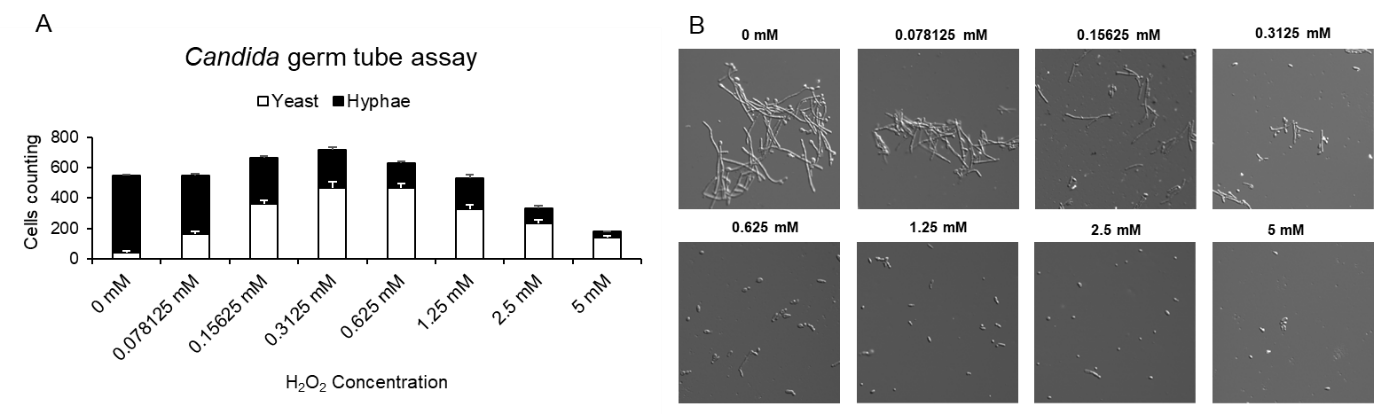
**

**Figure S3. H_2_O_2_ is a key factor affecting the morphological transition of *C. albicans.*** (A) After 3 hours of cultivation in FBS with different H_2_O_2_ concentrations, *C. albicans* was observed under an optical microscope, and area counts were performed using a hemocytometer to calculate the average value. (B) The morphological distribution of *C. albicans* was quantified by counting individual cells (yeast vs. hyphae) using a hemocytometer under an optical microscope. The ratio of yeast and hyphae in *C. albicans* was calculated based on the morphology.


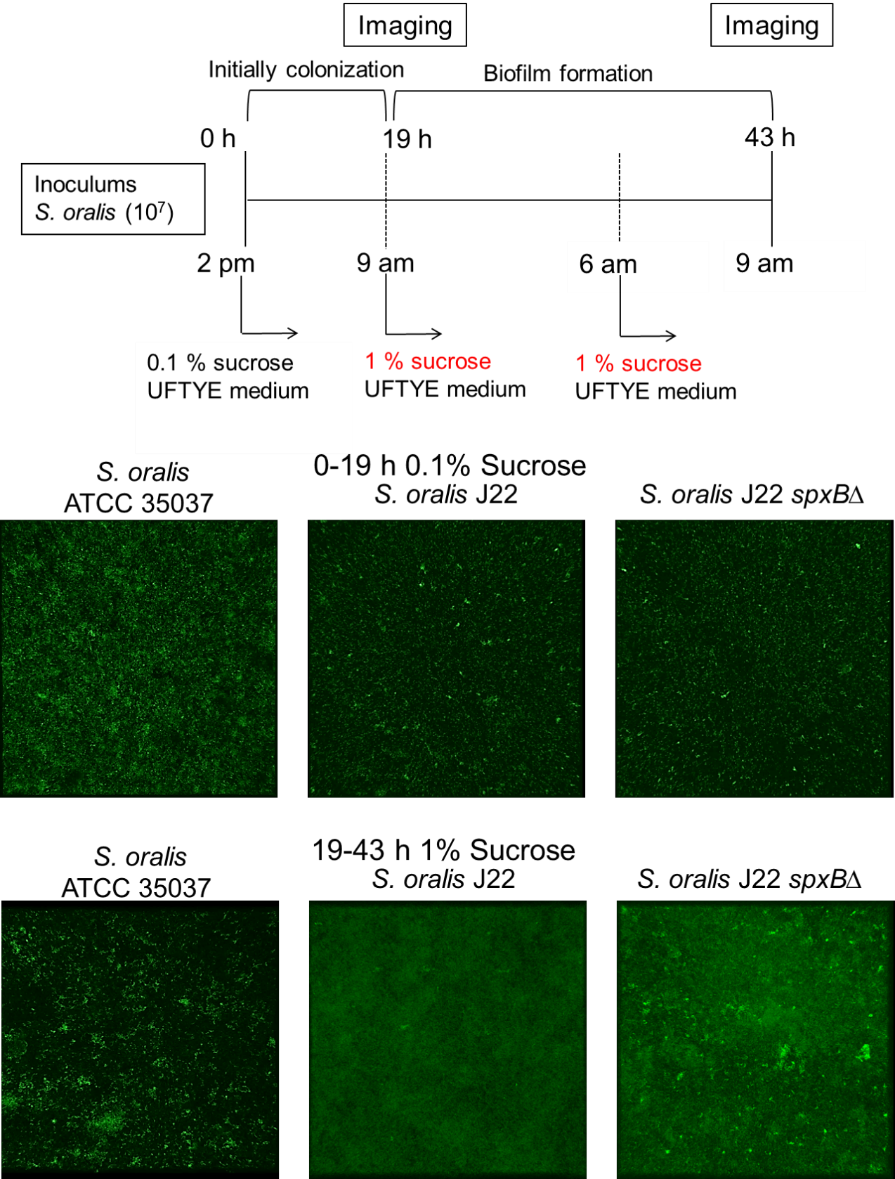


**Figure S4. Bacterial cell colonization of *S. oralis* at different development stages.** Different strains of commensal bacteria, with the same inoculum amount, were cultured in an ecological model for 19–43 h. The formation and attachment of single strains, including *S. oralis* ATCC 35037, *S. oralis* J22, and *S. oralis* J22 *spxB∆*, were visualized through confocal imaging.


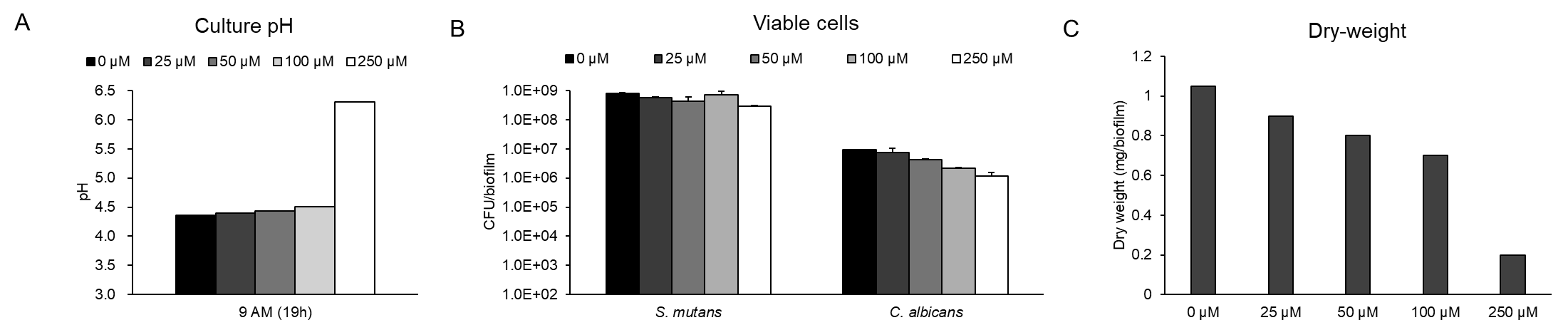


**F****igure S5**. **Effect of** **exogenous H₂O₂ on cross-kingdom biofilm formation.** Exogenous H₂O₂ at different concentrations (0–250 µM) was added at 0 h of cultivation. (A) After 19 h of cultivation, the pH value of the culture medium was measured across conditions with different H_2_O_2_ concentrations. (B) Biofilms were harvested and viable cell counts were determined. (C) Total biomass was determined by the dry weight of biofilms.


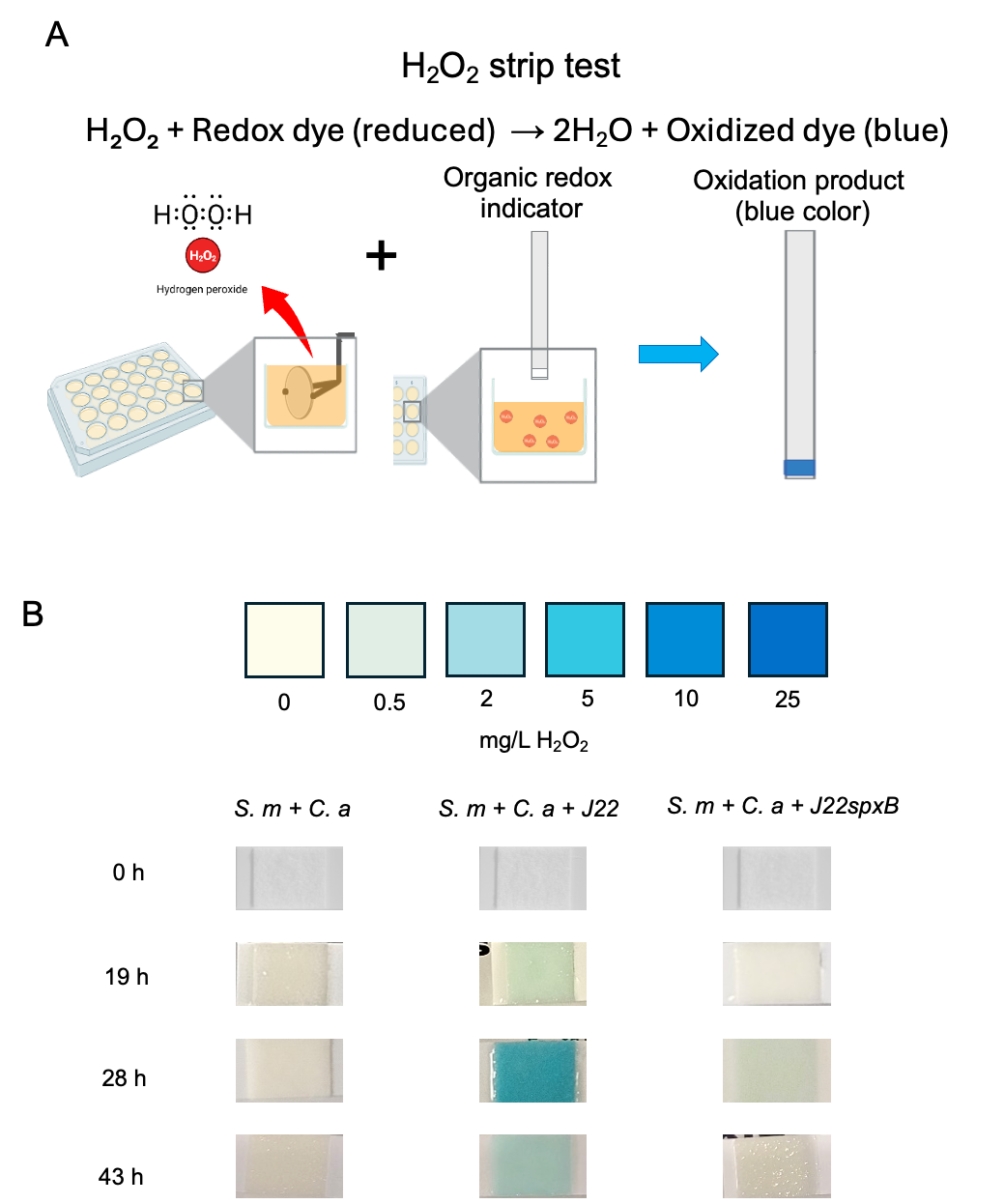


**Figure S6.** **H_2_O_2_ strips test of cross-kingdom culture medium*.*** (A) Steps and principles of H_2_O_2_-srtips color reaction in cross-kingdom biofilm culture medium. (B) Color reaction of H_2_O_2_-srtips in different groups in the early, middle, and mature stages of cross-kingdom biofilm culture medium.


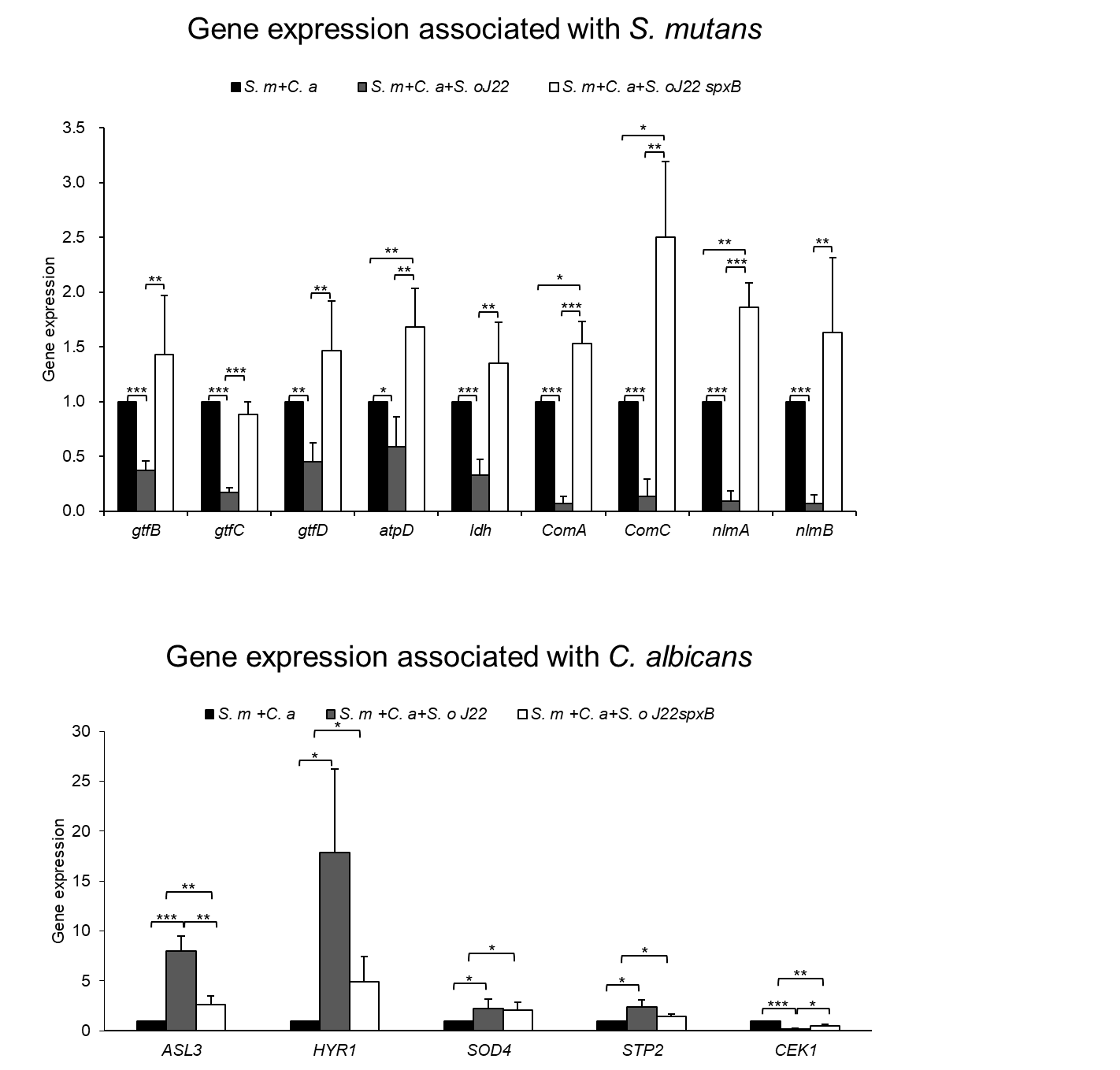


**Figure S7. Expression of genes associated with *S. mutans* and *C. albicans* in cross-kingdom biofilms.** The relative gene expression was assessed by comparing with the *S. mutans-C. albicans* biofilm group. The data were subjected to a *t*-test for pairwise comparison. *p < 0.05; **p < 0.01, ***p < 0.001.

**Table S1. Primer sequences related to targeted genes in *S. mutans* and *C. albicans***

|  | **Primer** | **Sequence (5′ to 3′)** | **Gene** | **Reference** |
| --- | --- | --- | --- | --- |
| ***S. mutans*** | 16S-F | ACC AGA AAG GGA CGG CTA AC | *16S rRNA* | Pfaffl MW. 2001. A new mathematical model for relative quantification in real-time RT-PCR. Nucleic Acids Res 29:e45. Wu J, Jiang X, Yang Q, Zhang Y, Wang C, Huang R. 2022. Inhibition of *Streptococcus mutans* Biofilm Formation by the Joint Action of Oxyresveratrol and *Lactobacillus casei*. Applied and Environmental Microbiology 88:e02436-21. |
|  | 16S-R | TAG CCT TTT ACT CCA GAC TTT CCT G |  |  |
|  | gtfB-F | AGC CGA AAG TTG GTA TCG TCC | *gtfB* | Xiao, J., Klein, M. I., Falsetta, M. L., ... & Koo, H. (2012). The exopolysaccharide matrix modulates the interaction between 3D architecture and virulence of a mixed-species oral biofilm. PLoS Pathogens, 8(4), e1002623. |
|  | gtfB-R | TGA CGC TGT GTT TCT TGG CTC |  |  |
|  | gtfC-F | TTC CGT CCC TTA TTG ATG ACA | *gtfC* | Xiao, J., Klein, M. I., Falsetta, M. L., ... & Koo, H. (2012). The exopolysaccharide matrix modulates the interaction between 3D architecture and virulence of a mixed-species oral biofilm. PLoS Pathogens, 8(4), e1002623. |
|  | gtfC-R | AAT TGA AGC GGA CTG GTT |  |  |
|  | gtfD-F | TAC CTT GGG CAC CAC AAC ACT | *gtfD* | Xiao, J., Klein, M. I., Falsetta, M. L., ... & Koo, H. (2012). The exopolysaccharide matrix modulates the interaction between 3D architecture and virulence of a mixed-species oral biofilm. PLoS Pathogens, 8(4), e1002623. |
|  | gtfD-R | TGC CGC CTT ATC ATC CTC ACT |  |  |
|  | atpD-F | CGT GCT CTC TCG CCT GAA ATA G | *atpD* | Xu X, Zhou XD, Wu CD. 2011. The tea catechin epigallocatechin gallate suppresses cariogenic virulence factors of *Streptococcus mutans*. Antimicrob Agents Chemother 55:1229-36. |
|  | atpD-R | ACT CAC GAT AAC GCT GCA AGA C |  |  |
|  | ldh-F | GGC GAC GCT CTT GAT CTT AG | *ldh* | Liu, Q., et al., Antimicrobial Peptide P-113-DPS Suppresses the Cariogenic Virulence of *Streptococcus mutans*. ACS Appl Bio Mater, 2025. 8(6): p. 4973-4980.  Xu X, Zhou XD, Wu CD. 2011. The tea catechin epigallocatechin gallate suppresses cariogenic virulence factors of *Streptococcus mutans*. Antimicrob Agents Chemother 55:1229-36. |
|  | ldh-R | GGT TAG CAG CAA CGA GGA AG |  |  |
|  | ComA-F | AGG GAC AGT TTC GTC ACT TAA TC | *comA* | L van der Ploeg, J.R., Regulation of bacteriocin production in *Streptococcus mutans* by the quorum-sensing system required for development of genetic competence. J Bacteriol, 2005. 187(12): p. 3980-9. |
|  | ComA-R | CCC ACA GTC TCT GGT ATC AAT C |  |  |
|  | ComC-F | GAC TGA TGA ATT AGA GAT TAT CAT TGG | *comC* | L van der Ploeg, J.R., Regulation of bacteriocin production in *Streptococcus mutans* by the quorum-sensing system required for development of genetic competence. J Bacteriol, 2005. 187(12): p. 3980-9. |
|  | ComC-R | TTT CCC AAA GCT TGT GTA AAA CT |  |  |
|  | nlmA-F | AAT GGA CAG CCA AAC ACT TTC | *nlmA* | Hossain, M.S. and I. Biswas, Mutacins from *Streptococcus mutans* UA159 are active against multiple streptococcal species. Appl Environ Microbiol, 2011. 77(7): p. 2428-34. |
|  | nlmA-R | TAA CAA GAG TCG CAC CTG CC |  |  |
|  | nlmB-F | TGT CAG AAG TTT TTG GTG G | *nlmB* | Hossain, M.S. and I. Biswas, Mutacins from *Streptococcus mutans* UA159 are active against multiple streptococcal species. Appl Environ Microbiol, 2011. 77(7): p. 2428-34. |
|  | nlmB-R | ACT CCA GCA CAT CCA GCA AG |  |  |
| ***C. albicans*** | DAD1-F | TTT CAT CTT CTG TAT CAG AGG AAC TTA TTT | *DAD1* | Thellin, O., et al. (1999). Housekeeping genes as internal standards: Use and limits. Journal of Biotechnology. |
|  | DAD1-R | ATG GGA TGA ATC ATC AAA CAA GAG |  |  |
|  | ASL3-F | CCA CTT CAC AAT CCC CAT C | *ASL3* | Green, C.B., et al., RT-PCR detection of *Candida albicans* ALS gene expression in the reconstituted human epithelium (RHE) model of oral candidiasis and in model biofilms. Microbiology, 2004. 150(2): p. 267-275. |
|  | ASL3-R | CAG CAG TAG TAG TAA CAG TAG TAG TTT CAT C |  |  |
|  | HYR1-F | ACT CCT ACT ACA TCA TCA AT | *HYR1* | Luo, G., et al., *Candida albicans* Hyr1p confers resistance to neutrophil killing and is a potential vaccine target. J Infect Dis, 2010. 201(11): p. 1718-28. |
|  | HYR1-R | GCA GAA CTA GAT TCA CTA AC |  |  |
|  | SOD4-F | TGA CTC CAA AGG CAA GGC ACC A | *SOD4* | Bink, A., et al., Superoxide Dismutases Are Involved in *Candida albicans* Biofilm Persistence against Miconazole. Antimicrobial Agents and Chemotherapy, 2011. 55(9): p. 4033-4037. |
|  | SOD4-R | TGG GCC AAC ACC TGA AGG CAA T |  |  |
|  | STP2-F | CCC CAA CAA CCA CCT TAT TTC | *STP2* | Böttcher, B., et al., The Transcription Factor Stp2 Is Important for *Candida albicans* Biofilm Establishment and Sustainability. Front Microbiol, 2020. 11: p. 794. |
|  | STP2-R | GAT GTT GTC GTT GTT GCT GC |  |  |
|  | CAP1-F | AGTCAATTCAATGTTCAAG | *CAP1*  (Candida AP-1) | Alarco AM, Raymond M. 1999. The bZip transcription factor Cap1p is involved in multidrug resistance and oxidative stress response in *Candida albicans*. J Bacteriol 181:700-8. |
|  | CAP1-R | AATGGTAATGTCCTCAAG |  |  |
|  | EFG1-F | GGT CAG TAT AAT GCT CCT GGT AAG | *EFG1* (Enhanced Filamentous Growth 1) | Glazier VE, Murante T, Murante D, Koselny K, Liu Y, Kim D, Koo H, Krysan DJ. 2017. Genetic analysis of the *Candida albicans* biofilm transcription factor network using simple and complex haploinsufficiency. PLoS Genet 13:e1006948. |
|  | EFG1-R | CAG CAC CAC CCT GGT AAT AAT |  |  |
|  | CEK1-F | TTAGAAATTGTTGGAGAAGGAGCAT | *CEK1* (Mitogen-Activated Protein Kinase) Pathway | Román E, Alonso-Monge R, Gong Q, Li D, Calderone R, Pla J. 2009. The Cek1 MAPK is a short-lived protein regulated by quorum sensing in the fungal pathogen *Candida albicans*. FEMS Yeast Research 9:942-955. |
|  | CEK1-R | GCAACTTTTTGTTGTGATGGTTTATG |  |  |
|  | RAS1-F | GGCCATGAGAGAACAATATA | *RAS1*  (cAMP-PKA Pathway) | Pang C, Chen J, Yang L, Yang Y, Qi H, Li R, Cao Y, Miao H. 2023. Shikonin Inhibits *Candida albicans* Biofilms via the Ras1-cAMP-Efg1 Signalling Pathway. Int J Gen Med 16:2653-2662. |
|  | RAS1-R | GTCTTTCCATTTCTAAATCAC |  |  |
